# How Ancestry Influences the Chances of Finding Unrelated Donors: An Investigation in Admixed Brazilians

**DOI:** 10.3389/fimmu.2020.584950

**Published:** 2020-11-06

**Authors:** Kelly Nunes, Vitor R. C. Aguiar, Márcio Silva, Alexandre C. Sena, Danielli C. M. de Oliveira, Carla L. Dinardo, Fernanda S. G. Kehdy, Eduardo Tarazona-Santos, Vanderson G. Rocha, Anna Barbara F. Carneiro-Proietti, Paula Loureiro, Miriam V. Flor-Park, Claudia Maximo, Shannon Kelly, Brian Custer, Bruce S. Weir, Ester C. Sabino, Luís Cristóvão Porto, Diogo Meyer

**Affiliations:** ^1^Laboratory of Evolutionary Genetics, Institute of Biosciences, University of São Paulo, São Paulo, Brazil; ^2^Instituto de Matemática e Estatística, Universidade do Estado do Rio de Janeiro, Rio de Janeiro, Brazil; ^3^Registro Nacional de Doadores Voluntários de Medula Óssea—REDOME, Instituto Nacional do Câncer, Ministério da Saúde, Rio de Janeiro, Brazil; ^4^Fundação Pró Sangue, Hemocentro de São Paulo, São Paulo, Brazil; ^5^Instituto Oswaldo Cruz, Fundação Oswaldo Cruz, Rio de Janeiro, Brazil; ^6^Departamento de Genética, Ecologia e Evolução, Instituto de Ciências Biológicas, Universidade Federal de Minas Gerais, Belo Horizonte, Brazil; ^7^Serviço de Hematologia, Hemoterapia e Terapia Celular, Hospital das Clínicas da Faculdade de Medicina da Universidade de São Paulo, São Paulo, Brazil; ^8^Fundação Hemominas, Belo Horizonte, Brazil; ^9^Fundação de Hematologia e Hemoterapia de Pernambuco, HEMOPE, Recife, Brazil; ^10^Hospital das Clínicas da Faculdade de Medicina da Universidade de São Paulo, Instituto da Criança, São Paulo, Brazil; ^11^Fundação Hemorio, Rio de Janeiro, Brazil; ^12^Epidemiology, Vitalant Research Institute, San Francisco, CA, United States; ^13^University of California San Francisco Benioff Children’s Hospital Oakland, Oakland, CA, United States; ^14^Department of Laboratory Medicine, University of California San Francisco, San Francisco, CA, United States; ^15^Department of Biostatistics, University of Washington, Seattle, WA, United States; ^16^Instituto de Medicina Tropical, Departamento de Moléstias Infecciosas e Parasitárias da Faculdade de Medicina da Universidade de São Paulo, São Paulo, Brazil; ^17^Laboratório de Histocompatibilidade e Criopreservação, Universidade do Estado do Rio de Janeiro, Rio de Janeiro, Brazil

**Keywords:** HLA, hematopoietic stem cell transplantation, genetic ancestry, admixture, MHC, Brazil

## Abstract

A match of HLA loci between patients and donors is critical for successful hematopoietic stem cell transplantation. However, the extreme polymorphism of HLA loci – an outcome of millions of years of natural selection – reduces the chances that two individuals will carry identical combinations of multilocus HLA genotypes. Further, HLA variability is not homogeneously distributed throughout the world: African populations on average have greater variability than non-Africans, reducing the chances that two unrelated African individuals are HLA identical. Here, we explore how self-identification (often equated with “ethnicity” or “race”) and genetic ancestry are related to the chances of finding HLA compatible donors in a large sample from Brazil, a highly admixed country. We query REDOME, Brazil’s Bone Marrow Registry, and investigate how different criteria for identifying ancestry influence the chances of finding a match. We find that individuals who self-identify as “Black” and “Mixed” on average have lower chances of finding matches than those who self-identify as “White” (up to 57% reduction). We next show that an individual’s African genetic ancestry, estimated using molecular markers and quantified as the proportion of an individual’s genome that traces its ancestry to Africa, is strongly associated with reduced chances of finding a match (up to 60% reduction). Finally, we document that the strongest reduction in chances of finding a match is associated with having an MHC region of exclusively African ancestry (up to 75% reduction). We apply our findings to a specific condition, for which there is a clinical indication for transplantation: sickle-cell disease. We show that the increased African ancestry in patients with this disease leads to reduced chances of finding a match, when compared to the remainder of the sample, without the condition. Our results underscore the influence of ancestry on chances of finding compatible HLA matches, and indicate that efforts guided to increasing the African component of registries are necessary.

## Introduction

Since the first allogeneic transplant in the late 1950s, there has been significant progress in the technical procedures and success rate of hematopoietic stem cell transplantation (HSCT). Nowadays, HSCT has become the standard treatment for several hematological diseases such as hematologic malignancies (leukemias, Hodgkin’s lymphoma), and also a curative treatment for some congenital or acquired disorders of the hematopoietic system (sickle cell anemia, severe aplastic anemia, thalassemia and inborn metabolism errors), as well as a therapeutic option in the treatment of some solid tumors (1, 2).

The success of allogeneic HSCT (allo-HSCT) is in part due to a better understanding of the role of HLA genes in the immune response. Classical HLA genes encode antigen-presenting proteins which are recognized by T-cell receptors. When HLA molecules bind a non-self antigen, cellular and humoral immune responses are triggered. Thus, in allo-HSCT the match between patient and donor HLA is critical, since different HLA alleles can generate distinct antigens detected as non-self during cellular immunological inspection. As a consequence, the patient’s immune system sees the donor cells with incompatible HLA as foreign and mounts an immune response, leading to rejection of the HSCT, and/or to graft versus host disease after grafting. Therefore, the gold standard for allo-HSCT is full compatibility between patient and potential donor at the 2 alleles of *HLA-A*, *-B*, *-C*, *-DRB1*, and *-DQB1* (10/10 match) [see review in Tiercy, 2016 (3)]. Nevertheless, in some cases mismatches may be allowed (9/10 match), especially at alleles which do not generate an anti-HLA antibody (4, 5), or when new therapeutic protocols of haploidentical transplant are used, in which the donor and recipient share a common HLA haplotype (6).

The extremely high number of alleles at HLA loci (more than 27,000 classical HLA alleles were described in the Immuno Polymorphism Database IMGT/HLA until July 2020, https://www.ebi.ac.uk/ipd/imgt/hla/stats.html) makes relatives of the patient the first option in searching for a donor. The patient’s probability of having the same HLA haplotypes as one of their siblings is 25%, and increases with the number of siblings (43.7% with 2, 57.8% with 3, 68.4% with 4 siblings, etc.) (7). However, according to the USA National Marrow Donor Program (NMDP), only 30% of allo-HSCT donors are chosen from close relatives, with unrelated allo-HSCT donors accounting for about 70% of cases (8). Therefore, public donor registries play a key role in meeting the demand for unrelated donors.

According to the World Marrow Donor Association (WMDA), which includes 53 associated countries, the number of registered unrelated donors exceeds 35 million individuals and 700,000 cord blood units (WMDA, 2019; https://statistics.wmda.info/). Despite this large number, a major concern has been to understand how representative existing registries are of the general population, since the high diversity of HLA loci, their geographical heterogeneity (9, 10), and biases in volunteer recruitment, can result in certain groups within the population having reduced access to donors.

This scenario may be critical in the case of admixed populations, which trace their ancestry to different geographic regions, and has been the focus of several recent studies on how ethnicity influences the probability of finding a donor (8, 11–13). In the USA, an NMDP study found that while 75% of patients who self-identify as White-European found a donor with 7/8 HLA matching, only 16% for African-Americans found a match (8). A Memorial Sloan Kettering Cancer Center study of 7/8 or 8/8 HLA matching found that 78% of patients with Northwestern European ancestry found a donor, while this number fell to 44% for those with South European ancestry and 22% for those with African ancestry (13). Theoretical studies by Bergstrom et al. (11, 14) also found that the probability of not finding a match in the NMDP is highest among African-Americans, when compared to other groups. These findings have been interpreted as an outcome of a combination of factors, including a lower representation of African-Americans in databases, as well as the greater genetic diversity of populations originating in Africa, which decreases the likelihood that two unrelated individuals will share a multi-locus HLA genotype (15).

While the studies discussed above make a convincing case regarding the impact of self-identification upon the chances of finding a donor, they do not address the low correspondence between self-identification and genetic ancestry (16–18). In admixed populations, individuals who self-identify to the same group frequently have extremely variable genetic ancestries. This is expected, since self-identification involves a complex interplay of social and genealogical components (19) and varies among geographic regions in Brazil (20, 21).

**Figure 1:**
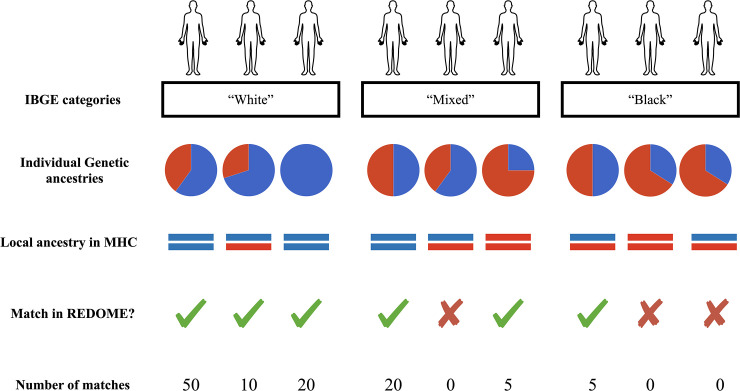
Schematic representation of the strategy used in this study. Individuals are classified by three criteria: “IBGE categories” are self-identified categories defined by the Brazilian Institute of Geography and Statistics (IBGE), based on phenotypic characteristics such as skin color, and can be “Black”, “Mixed”, “Indigenous”, “White”, and “Yellow”; “Genetic Ancestry” is inferred with genetic markers spread throughout the genome, and describes the proportion of an individual’s genome that can be assigned to a continent of origin (with red and blue corresponding to African and European proportion ancestries respectively); “MHC ancestry” is the genetic ancestry of the segment of chromosome 6 including the MHC region within an individual (again, with red and blue corresponding to African and European ancestries). For each individual, classified with these criteria, we queried REDOME for the presence of a matching genotype and recorded the number of matches, when they were found.

Here, we investigate the role of genetic ancestry in determining the chances that an individual will find a match in REDOME (*Registro Nacional de Doadores Voluntários de Medula Óssea*), the Brazilian Marrow Donor Registry. We investigate how three layers of information affect the chances of finding an HLA match: (i) the self-identification; (ii) the genetic ancestry; (iii) the genetic ancestry of the MHC region (**Figure 1**). Brazil harbors the largest number of individuals with African ancestry outside Africa (https://www.slavevoyages.org/) and has one of the most admixed populations in the world. In addition, REDOME is the third largest marrow registry in the world, with more than 5 million registered individuals (as of July 2020). We use samples from two cohorts of Brazilians for which we have information on self-identification, and that were previously genotyped with high density SNP arrays (22, 23), providing a total of 8,037 individuals. For each individual we estimate their genetic ancestry, their ancestry within the MHC, and we impute their HLA genotypes using the SNPs flanking the classical HLA loci. We then query the REDOME to identify how many matches are present for each individual, and use these results to compare how different measures of ancestry influence the chances of finding a match (**Figure 1**).

## Material and Methods

### Datasets

Individuals from two Brazilian cohorts were queried for matches in REDOME, as described below. Although only a subset of individuals in these cohorts are in fact patients for whom there is an indication of transplantation, for the purposes of our analyses we will treat all samples as patients, given that our goal is to establish relationships between ancestry and chances of finding a potential donor among Brazilians with varying ancestries.

The Brazilian National Ethical Committee for Research (CONEP, resolution 15.895 and resolution 1.297.627), local ethics committees at each participating center, the Institutional Review Boards at the University of California, San Francisco and the REDS-III data coordinating center, RTI International, all reviewed and approved the study.

### The Recipient Epidemiology and Donor Evaluation (REDS)-III Brazil Sickle Cell Disease (SCD) Cohort

The REDS-III Brazil SCD cohort was established to study the epidemiology and transfusion outcomes of SCD in Brazil, and includes 2,795 individuals recruited between 2013 and 2015 in 4 reference centers: Fundação Hemominas (Belo Horizonte, Juiz de Fora, and Montes Claros), Fundação Hemope (Recife), Fundação Hemorio (Rio de Janeiro), and Instituto da Criança Hospital das Clínicas da Faculdade de Medicina da Universidade de São Paulo (São Paulo) (**Figure S1**). Samples were previously genotyped with a high density SNP array (Axiom Transfusion Medicine Array, TM Array, Affymetrix, Santa Clara, CA, USA) and are available in the dbGAP (phs001972.v1.p1). After filtering (calling < 97%, Hardy-Weinberg p-value > 10^-8^, sample missing data <5%) the dataset had 2,703 individuals and 820,837 SNPs (22).

### EPIGEN Brazil Initiative

The Brazilian EPIGEN Initiative (EPIGEN) is the largest Brazilian resource (n=6,487) for population genomics. EPIGEN includes three cohorts: Salvador (n=1,309), from northeast Brazil (24); Bambuí (n=1,442), from the southeast (25); and Pelotas (n=3,736), from south Brazil (26) (**Figure S1**). The samples were previously genotyped with the HumanOmin 2.5 (Illumina, San Diego, CA, USA) SNP array (23) and are available in the European Nucleotide Archive (PRJEB26388 (ERP108374)), under EPIGEN Committee Controlled Access mode. We selected samples for which both genotype data and self-identification were available, resulting in a total of 5,334 samples (Salvador, n = 918; Bambuí, n = 765; and Pelotas, n = 3,651).

### HLA Imputation

For admixed and non-European populations, Attribute Bagging is the most accurate approach for imputing HLA alleles from SNP data (27–29). Here we use the HLA Genotype Imputation with Attribute Bagging (HIBAG) R package (30). HIBAG uses a reference panel with data on both HLA alleles and SNPs in the MHC region to infer HLA alleles for samples with only SNP data.

We built a multi-ancestry imputation model for *HLA-A, -B, -C, -DQB1*, and *-DRB1* with two-field level of resolution using the 1000 Genomes phase III (2,504 individuals) as a reference panel (31, 32). We selected SNPs within the MHC region which are present in both the TM array (10,711 SNPs) and HumanOmin2.5 (9,187 SNPs). For each SNP array, HIBAG models were built using SNPs within 500 kb flanking each HLA locus, and 100 bootstrap samples as classifiers. Out-of-bag estimated accuracies for each model are reported in **Table S1**, and models are available upon request. HLA imputation was performed separately for REDS-III and EPIGEN, and the posterior probability estimated by the model was used as a confidence score, allowing inference of the predicted accuracy of the imputation for each HLA genotype. We used the empirical cumulative distribution function (ECDF) to compare the posterior probability distribution of the imputed HLA genotypes among IBGE categories and genetic ancestries (**Tables S2 **and** S3** and **Figures S6 **and** S7**).

Since Native American populations are not well represented in the reference panel, HLA imputation for individuals of this ancestry is uncertain, so we chose to focus exclusively on the effects of African and European ancestry in subsequent analyses. Understanding of how Native American ancestry impacts the chances of finding a donor in REDOME will require direct typing of HLA alleles.

### Finding Matches in REDOME

The Brazilian Bone Marrow Donor Registry (REDOME) was established in 1993, funded by the Brazilian Ministry of Health. As of April 30, 2019, REDOME had 4,869,224 registered volunteers. All individuals are genotyped at *HLA-A, -B* and *-DRB1*, and a subset also at *HLA-C* (n=125,248) and *-DQB1* (n=123,298), with HLA resolution ranging between low (e.g., serological assays) to medium/high (e.g., SBT-PCR, Next Generation Sequencing) (**Table S4**).

For each individual, we queried REDOME for potential donors at both low and medium resolutions (one and two HLA allele fields, respectively). At low-resolution an allele such as A*34:02 is compatible with those from donors carrying any variant of A*34 (e.g., A*34:01, A*34:02, etc.). At medium-resolution, only variants of A*34:02 (for example A*34:02:01G) are considered compatible, and any NMDP allele code containing the 34:02 allele.

We searched REDOME for full matching at three, four and five HLA loci, hereinafter referred to as 6/6, 8/8 and 10/10, respectively. Searches for 6/6 matching were for *HLA-A*, *-B* and *-DRB1*; 8/8 searches further include *HLA-C*; 10/10 searches further include *HLA-DQB1*. We also carried out analyses that allowed mismatches at a single allele (5/6, 7/8 and 9/10).

To evaluate the impact of the predicted accuracy of imputation on our results, we also performed searches on a subset of individuals with posterior probability of HLA imputation greater than 0.8 at all surveyed loci (2,554 individuals in total; REDS, n = 914; Bambuí, n = 433; Salvador, n = 219; Pelotas, n = 988). In the main text, all results refer to the dataset of 8,037 individuals, without filters for predicted accuracy of HLA imputation, and we refer to **Supplementary Materials** for results on the high confidence subset when appropriate.

### Inference of Genetic Ancestry

To infer the genetic ancestry for Brazilians, we used the three parental populations: 502 African and 503 European individuals from the 1000 Genomes Project phase-III (33), and 234 Native American individuals (34).

Genetic ancestry for each Brazilian individual was estimated with ADMIXTURE v.1.23 (35), which uses a maximum likelihood framework, based on multilocus SNP genotypes. We performed a supervised analysis with K=3 (corresponding to African, European and Native American ancestries), 2000 bootstrap replicates, windows of 50kb and step size of 10kb, and LD R^2^ threshold of 0.1.

To infer the genetic ancestry of the MHC region for each individual, we used RFMix v.2 (36). First, genotypes for chromosome 6 were phased with the SHAPEIT v.2.12 software (37). Parental populations were subsampled to have equal sample sizes (234 individuals). We ran RFMix with default parameters, a time since admixture of 8 generations, and 2 EM iterations. Local ancestry was estimated for the whole of chromosome 6, but we subsequently focused on the ancestry of the subset of the MHC region encompassing the classical HLA loci, delimited by *HLA-A* and *-DQB1*, and spanning 2,724,220 bp.

### IBGE Categories, Genetic Ancestry and the Chance of REDOME Match

The Brazilian Institute of Geography and Statistics (*Instituto Brasileiro de Geografia e Estatística*, IBGE) defines a widely used classification, which identifies individuals as “Black” (*Preto*), “Mixed” (*Pardo*), “Indigenous” (*Indigena*), “White” (*Branco*) and “Yellow” (*Amarelo*), creating categories that confound skin color, social self-identification and genealogy (38). Despite critiques (39), this classification remains used in epidemiological studies, the Brazilian census, blood centers, and by REDOME. All individuals in the REDS-III and EPIGEN cohorts, as well as those in the REDOME database, are self-identified according to these categories. Here, we use only “Black”, “Mixed” and “White”, since “Indigenous” and “Yellow” contribute to only 1.8% of our sample (**Figures S2 **and** S3**). Our use of these IBGE categories does not assume they are natural groupings with a biological basis. Rather, our goal is to examine how this widely used classification predicts the chances of finding a match in REDOME, and how results based on this classification compare to those obtained when groups defined by genetic ancestry are used.

We used a univariate logistic regression model, assuming “match in REDOME” (yes/no), as the outcome and IBGE categories or genetic ancestry as predictors, computing the odds ratio for each contrast. For genetic ancestry (estimated as percentages of African, European and Native American ancestries per individual), individuals were classified into quartiles of increasing African ancestry (Q1, Q2, Q3, Q4). For local ancestry analyses, we identified three genotypic classes for the MHC region in each individual: African/African, African/European or European/European. Because the ancestries in the MHC have a trimodal distribution (**Figure S5**), we could classify individuals into 3 groups according to number of chromosomes with African MHC: 0, 1 and 2 (rounding intermediate values, resulting from chromosomes with mixed ancestries, to the nearest integer).

## Results

### IBGE Categories Explain Only a Small Amount of Genetic Ancestry

According to IBGE census (2019) (40) the Brazilian population has composition of 45.22% “White”, 45.06% “Mixed”, and 8.86% “Black”, 0.47% “Yellow” and 0.38% “Indigenous”, whereas REDOME’s composition is 54.64% “White”, 23.44% “Mixed”, 7.17% “Black”, 0.46% “Indigenous”, 3.31% “Yellow” and 10.97% “Non Informed”, indicating a deficit of the “Mixed” category in REDOME with respect to the Brazilian population as a whole.

The REDS-III and Salvador (EPIGEN) cohorts have high proportions of “Mixed” and “Black” and high African ancestry, whereas Bambuí and Pelotas are predominantly “White” and have high European ancestry (**Table 1**). The merged dataset has 44.1% “White”, 32.6% “Mixed” and 19.8% “Black” individuals, and the average genetic composition was 62.4% European, 30.9% African and 6.1% Native American.

**Table 1:** Brazilian Institute of Geography and Statistics (IBGE) categories and genetic ancestry across cohorts.*

Dataset	IBGE categories (%)						Genetic Ancestry (%)		
	Black	Indigenous	Mixed	White	Yellow	Non Informed	African	European	Native American
REDS-III	26.7	0.5	58.6	10.7	0	3.5	49.7	43.6	6.7
EPIGEN Salvador	25.4	0.1	61.9	7.5	0	5.1	50.3	43.8	5.9
EPIGENBambuí	6.3	0	34.4	59.3	0	0	14.2	79.4	6.0
EPIGEN Pelotas	16.1	1.8	5.6	74.8	1.7	0	15.5	77.3	7.2
Merged Dataset	19.8	1	32.6	44.1	0.8	1.7	30.9	62.4	6.1

*Percentages refer to the proportion of individuals in each IBGE category, or the average per individual of each genetic ancestry.

**Figure 2:**
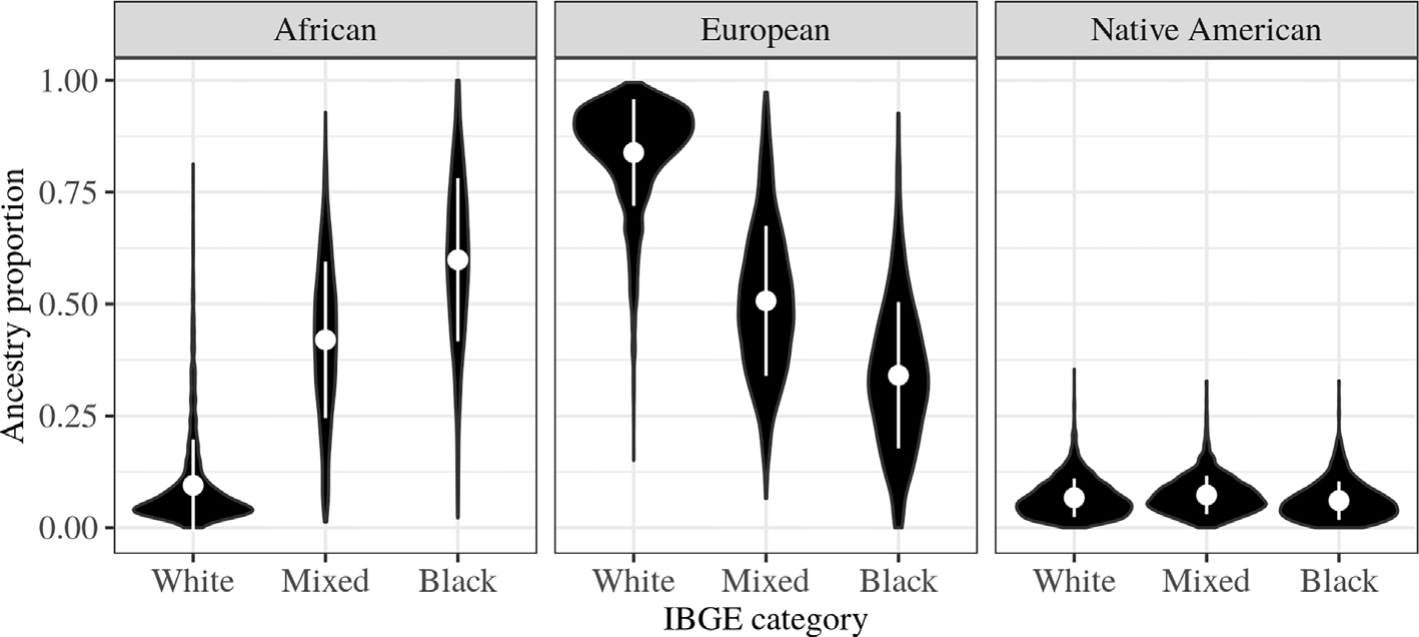
Relationship between Brazilian Institute of Geography and Statistics (IBGE) categories (“Black”, “Mixed”, and “White”) and genetic ancestry (African, European and Native American) estimated using ADMIXTURE software in the merged dataset (REDS-III + EPIGEN). The white circles represent the averages and the vertical white lines correspond to one standard deviation, and the black shapes describe the distribution of ancestries within each IBGE category.

We initially examined the relationship between IBGE categories and genetic ancestry (**Figure 2**). Individuals categorized as “Black” have, on average, greater African genetic ancestry than those categorized as “White” (ANOVA p-value < 2x10^-16^; Tukey test p-value < 0.00001). Despite the statistical significance, much of the variation in ancestry among individuals is not captured by the IBGE categories, with about 36% of the variation in African and European genetic ancestry not being explained (r^2^ = 0.64; **Figure S4**). The poor correlation between self-identified categories and genetic ancestry is even more apparent for the REDS and Salvador cohorts, both of which have a high proportion of individuals in the “Black” and “Mixed” categories (**Table 1**), and in which most of the variation in genetic ancestry is not captured by the IBGE categories (r^2^ = 0.31).

Our finding of a weak correlation between IBGE categories and genetic ancestry is consistent with previous studies of admixed Brazilians (18, 41), and suggests that the higher the admixture proportion, the lower the correlation between them. This suggests that there may be important differences between the influence of IBGE categories and of genetic ancestry on the chances of finding a match in REDOME, and it is this question we address in subsequent next sections.

### The Success of Finding Matches in REDOME

The proportion of individuals in the merged dataset for whom we find at least one match in REDOME at low resolution was 87.7% (6/6), 15.1% (8/8), and 13.0% (10/10). For medium resolution, matches were found for 51.3% (6/6), 6.1% (8/8), and 2.0% (10/10) of the individuals (**Table 2**).

**Table 2:** Proportion of individuals with at least one match in Brazilian Bone Marrow Donor Registry (REDOME), median number of matches and the maximum number of matches at low and medium resolution.

Loci	Low resolution			Medium resolution		
	% at least one match	Median number of matches	Maximum number of matches	% at least one match	Median number of matches	Maximum number of matches
6/6	87.7	15	3,157	51.3	5	2,229
8/8	15.1	3	81	6.1	2	56
10/10	13.0	3	79	2.0	3	49

The total number of potential donors per individual varies depending on the number of loci queried and resolution (**Table 2**). Among those individuals who find at least one match, the median number of potential donors at low resolution is 15 (6/6), 3 (8/8), and 3 (10/10); at medium resolution the values were 5 (6/6), 2 (8/8), and 3 (10/10).

Due to the small number of matches for medium resolution (and low statistical power to investigate the effect of ancestry), we subsequently only report results for low resolution matching. The results for all HLA combinations of low/medium resolution and numbers of loci are available in the **Supplementary Material** (**Figures S8–S16**).

### Self-Identification as “Black” and High African Ancestry Correlate With Lower Chances of Finding a Match

**Figure 3:**
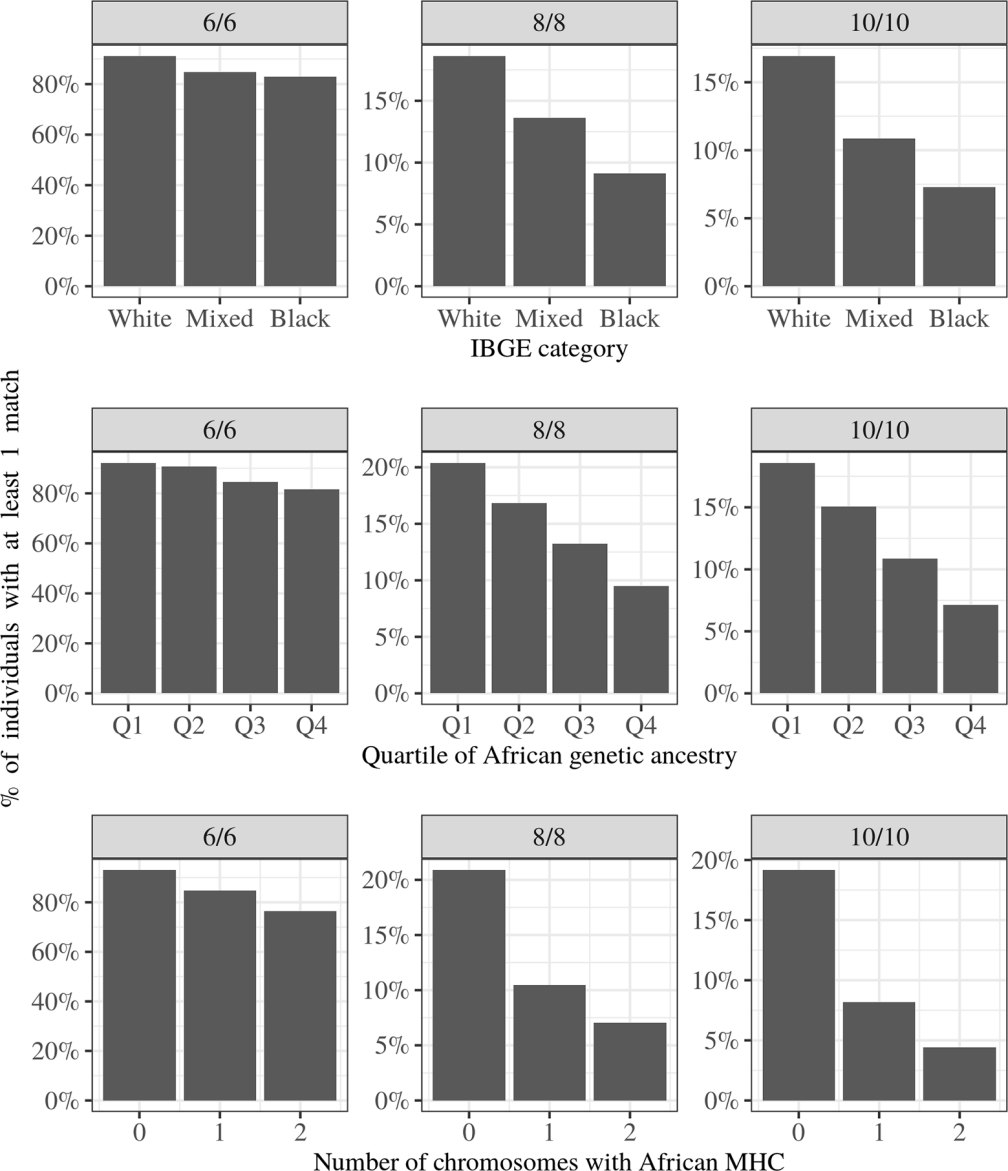
Percentage of individuals from the merged dataset with at least one match in Brazilian Bone Marrow Donor Registry (REDOME) for low resolution 6/6, 8/8, and 10/10. Top row: Brazilian Institute of Geography and Statistics (IBGE) categories; Middle row: Quartile of African ancestry; Bottom row: Number of chromosomes with African MHC per individual.

We first quantified the proportion of individuals with at least one match in REDOME (**Figure 3**, top row). In the 6/6 queries, 91.1% of the individuals classified as “White” find at least one donor compared to 84.7% and 82.9% of those categorized as “Mixed” and “Black”, respectively. In the 10/10 queries 16.9% of “White” individuals find a match, while only 7.3% of “Black” individuals do.

When we compare the chances of finding a match across groups defined by their proportion of African genetic ancestry, a similar pattern emerges, with the chance of finding a match decreasing as African genetic ancestry increases (**Figure 3**, middle row). For 6/6 queries, 92.1% of the individuals in the first quartile (with less than 0.61% African genetic ancestry) find at least one potential donor, as opposed to 81.7% of those in the fourth quartile (more than 51.31% African genetic ancestry). Again, in the 10/10 queries the difference is more extreme (18.6% vs. 7.12%).

While genetic ancestry refers to an average of the entire genome, it is possible to assign an ancestry specifically to the MHC region. We found that the greater the African ancestry in the MHC, the lower the chances of finding at least one potential donor. The percentage of individuals with 0 versus 2 chromosomes with African MHC who find a match is 93.0% vs. 76.5% at 6/6, and 19.2% vs. 4.4% at 10/10, respectively (**Figure 3**, bottom row).

Using a univariate logistic regression, all comparisons between groups with least African ancestry (i.e., “White” or Q1 of African ancestry) and most African ancestry (i.e., “Black” or Q4 of African Ancestry) are significant (p-value < 2×10^-16^; see **Supplementary Material Tables S5 **and** S6** for complete set of OR).

Among individuals with at least one match, the number of potential donors varies substantially (**Table 2**). We therefore assessed how the IBGE categories, genetic ancestry and MHC ancestry influence the number of potential donors found. Individuals categorized as “Black” and those with great African genetic ancestry, on average have a smaller number of potential donors, as compared to those categorized as “White” or having less African ancestry (**Figures S11–S13**). For the 6/6 queries, these differences are significant for all layers of ancestry (IBGE categories, African genetic ancestry, and MHC ancestry; p-value < 0.05, Mann-Whitney U test, **Table S7**).

Therefore, ancestry affects the chances of finding a match in two ways. First, in comparison with individuals categorized as “White” or having more European ancestry, a lower proportion of individuals classified as “Black” or carrying higher African ancestry find a match in REDOME (**Figure 3**). Second, among those who do find a donor, those classified as “Black” or having a greater African ancestry, on average have a smaller number of potential donors than “White” or genetically more European individuals (**Figures S11–S13**).

### Matching of Ancestries Between Donors and Recipients

Overall, our results support that ancestry influences the chances of finding a match in REDOME. Thus, we next asked whether the ancestry of an individual is the same as that of his/her potential donors. Since we do not have genetic ancestry data for REDOME, we analyze IBGE categories.

**Figure 4:**
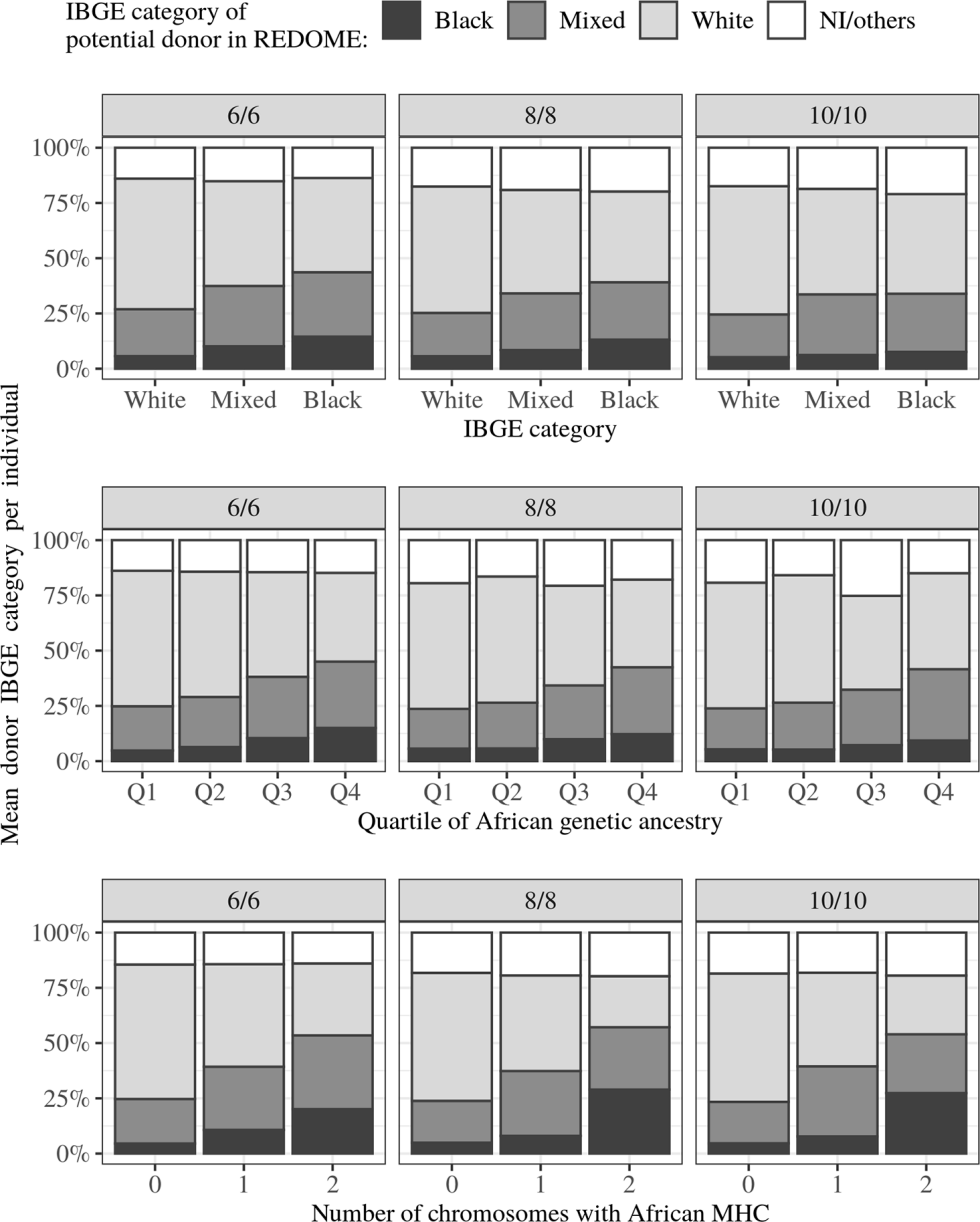
Mean frequency of Brazilian Institute of Geography and Statistics (IBGE) category of the potential donors for individuals in different IBGE categories, African ancestry quartiles, or with different numbers of chromosomes with African MHC. For each individual we calculate the average IBGE category of his/her potential donors, then for each category or genetic ancestry group we computed the group-level average (average of individual averages). Individuals who are “Black” or have more African genetic ancestry find proportionately more donors in the same category/ancestry.

For individuals with at least one match, we computed the average proportion of potential donors from each IBGE category. We find that individuals in the “Black” IBGE category, as compared to those in “White” and “Mixed”, match proportionally more to “Black” potential donors (5.7%, 10.1%, and 14.5% of “Black” donors for “White”, “Mixed” and “Black” individuals, respectively, at 6/6) (**Figure 4**, top row). When considering genetic ancestry, individuals with progressively more African ancestry match proportionately more to “Black” potential donors (4.8%, 6.4%, 10.4% and 15% of “Black” donors for individuals in Q1, Q2, Q3, Q4 at 6/6) (**Figure 4**, middle row). This trend is more pronounced when we consider ancestry in the MHC, where individuals who carry two chromosomes with African MHC match a pool of donors which is 20% “Black”, versus 4.5% for individuals with no chromosomes with African MHC (results for 6/6 matching) (**Figure 4**, bottom row).

### Finding Donors for Sickle Cell Disease Patients

Within our merged dataset, the individuals who are from the REDS-III cohort are diagnosed as having sickle cell disease (SCD). We therefore repeated our previous analyses on these individuals, so as to assess the influence of ancestry for a set of individuals who are eligible for HSTC (42).

Sickle Cell Disease (SCD) is a genetic disorder with an African origin, affecting 3,500 newborns annually, and estimated to affect between 25,000–30,000 people in Brazil (43). The only curative treatment available is HSCT. Although transplantation among family members is recommended, parents or siblings may be carriers of the sickle cell trait, which compromises them as donors. Thus, unrelated allo-HSCT transplantation or the haploidentical transplant, are useful options.

Based on pre-established criteria by the Brazilian Ministry of Health, Flor-Park and col. (42) identified a subset of 417 patients within REDS-III who are eligible for transplantation. This subset consists of 30.3% “Black”, 54.7% “Mixed”, 10.3% “White” individuals, with 4.8% in other IBGE categories. The average genetic ancestry of these individuals is 52.0% African, 41.9% European and 6.1% Native American.

Among these SCD patients, 8.1% find a match in REDOME (10/10 low resolution), compared to 14.4% among individuals without SCD in the EPIGEN cohort (z-score=−3.563, p-value= 0.0004) (**Tables S9 **and** S10**). Thus, consistent with the previous analyses, we show that individuals with increased African ancestry – in this case a set of patients with SCD and an indication for transplantation – have a decreased chance of finding a match in REDOME when compared to individuals from a group which has lower African ancestry (**Tables S8-S10**).

## Discussion

We investigated how ancestry is related to an individual’s chance of finding a match in REDOME, the third largest bone marrow registry in the world. For a sample of 8,037 Brazilians, we used three approaches to assess ancestry: (i) self-identified categories (e.g., “Black”, “White”, or “Mixed”), (ii) genetic ancestry (i.e., the percentage of an individual’s genome which is African or European; **Figure S4A**), and (iii) genetic ancestry specific to the MHC region (i.e., how African or European are an individual’s chromosomes within the MHC region; **Figure S4B**).

### A Hierarchy of Effects: Self-Identification, Genome-Wide Genetic Ancestry, and Genetic Ancestry in the MHC

As in other studies (17, 44, 45), we find that individuals categorized as “Black” or “Mixed” have a lower chance of finding a potential donor than “Whites”. We also show that African genetic ancestry, both genome-wide and within the MHC, is associated with a marked reduction in the chances of finding a match. To directly compare the differences in chances of finding a match between classes such as “Black” and “White’’ to those between extremes of African ancestry, we performed an additional analysis, in which we matched the size of groups being compared (**Table 3**, explanation in legend). We document a hierarchy of effects of African ancestry on the chances of finding a match, with the chances progressively decreasing from IBGE categories, to genomic ancestry, and reaching a maximum decrease when comparing classes based on ancestry in the MHC region.

**Table 3:** Percent decrease in the chances of finding at least one match for “Black” or most genetically African individuals, relative to “White” or least genetically Africans, respectively.*

Query(low resolution)	Black vs. White	Most vs. Least African (genome)	Most vs. Least African (MHC)
6/6	9%	11%	18%
8/8	51%	54%	65%
10/10	57%	60%	75%

*All contrasts are referenced to that between “Black” (n=1,589) and “White” (n=3,544) sample sizes, involving a partition of ancestry of 3,544 individuals into two groups.

The association between African ancestry and decreased chances of finding a match has previously been explained as a consequence of the higher polymorphism of African populations (15). Humans originated in Africa, and spread over the world experiencing a series of bottlenecks (46, 47), which caused non-Africans to have lower genetic diversity. This reduced variation in non-Africans extends to HLA loci (9, 48, 49), and as a consequence the chance that two unrelated Africans will share a multi-locus HLA genotype is lower than that seen between two Europeans. In this study, we show that African ancestry in the MHC region is the strongest predictor of decreased chances of finding a match in REDOME. Our interpretation is that the IBGE categories and genetic ancestry genome-wide capture information about the ancestry within the MHC, thus accounting for their effects on the chances of finding a match. However, the ancestry within the MHC region is most directly associated with the chances of finding a match, since it captures information about the ancestry of the HLA allele an individual carries, which drives the chances of finding a match.

In addition to the role of African ancestry on the chances of finding a match, admixture in Brazilians may also be an important factor. The EPIGEN cohort (used in the present study), identifies that populations from different regions of the African continent contributed to the Brazilian gene pool (50), so that populations that never met in the African continent may experience gene flow in Brazil. If we transpose this issue to the MHC region, new HLA genotypic combinations may emerge among individuals of African ancestry, adding to the difficulty in finding unrelated voluntary donors. New genotypic combinations created by admixture may also be a more general issue, regardless of ancestry. For example, patients who do not find donors frequently carry alleles which are present in REDOME, but for which the genotype combination was not found (51). Thus, future studies should also model and quantify the role of admixture in shaping the chances of finding a match in REDOME.

### Clinical Implications

Our dataset contains a group of patients with sickle cell disease (SCD) and a recommendation for transplantation. At low resolution, 8.1% of SCD patients surveyed find a 10/10 match, compared to 14.4% for those without SCD (in the EPIGEN cohort; p-value=0.0004). This provides a concrete example of how patients with a higher than average African ancestry, when seeking transplantation, have reduced chances of finding a match.

There are still few studies with HSTC from unrelated donors for SCD patients [review in Oevermann and Sodani, 2020 (52)]. This scarcity is due to the difficulty of finding a match with unrelated donors in international public registries (52, 53). As a consequence, many of these unrelated allo-HSTC were performed with some level of HLA mismatch and were associated with a high prevalence of engraftment failure as well as graft-versus-host disease (53, 54). Recently, important advances have been reported, both with improved match between unrelated patients and donors (HLA high resolution full match or haploidentical) and in treatment with post-transplant drugs (55). However, these studies are not sufficient to guarantee the safety of this treatment (52), which is still evaluated as experimental. For this reason, HSCT between siblings is the gold standard for patients with SCD, and in Brazil it is the only modality recommended by the Ministry of Health (ordinance SAS/SCTIE n° 5/2018) with support from the Public Health System (Sistema Único de Saúde - SUS). Therefore, despite the curative potential of unrelated allo-HSCT for many hematological malignant diseases, it is not yet used routinely in clinical treatment of SCD. This is explained, to a large degree, by the high African genetic ancestry in patients with SCD, which decreases the chance of finding a compatible donor. As a consequence, the small number of transplants performed from unrelated donors have often allowed mismatches.

The drawbacks associated with the impossibility of locating HLA-compatible donors for HSCT are potentially severe. In the case of some hematological malignant diseases, such as acute lymphoblastic leukemia in adults, a lack of a HLA-compatible donor may mean withdrawing the therapeutic cornerstone, which is HSCT, and consequently compromising patient survival (56). This scenario is similar for other important non-malignant diseases, i.e. aplastic anemia, in which the HSCT plays a pivotal therapeutic role. However, it seems even more complicated in the case of SCD patients, for whom the low chance of finding a compatible unrelated donor makes it difficult to gather large samples to evaluate appropriate post-transplant parameters and treatment options. Therefore, at the moment, most SCD patients who are eligible for transplantation would not benefit from the positive effects of unrelated allo-HSCT, including decrease of vaso-occlusive episodes, donor-derived erythropoiesis and restoration/stabilization of function of damaged organs (57).

### HLA Diversity and Donor Recruitment Challenges

Previous studies have shown that individuals with the same ancestry tend to share HLA alleles more often than those from different populations (12, 58). Our study confirms this trend, with the number of “Black” donors increasing with the recipients’ degree of African ancestry (**Figure 4**). On the other hand, many potential donors for “Black” individuals are in fact “White”. This is expected in an admixed population for several reasons: many HLA alleles are shared around the world; REDOME is predominantly “White” and “Mixed”; self-identified categories do not accurately capture the genetic ancestry; potential “White” donors may contain African ancestry in the MHC region.

Faced with this complex scenario, it is natural to ask how large a registry should be, in order to adequately cover the diversity of a population. Expanding the size of the registry indefinitely is not an economically viable option, so strategies have been proposed to define an “optimal registry size”. These efforts model the frequency of the most common HLA haplotypes, the distribution of individuals among regions within a country, and probability of matching (59–62). This task is particularly complex in admixed populations, since genetic diversity varies among groups.

As is the case for the NMDP (11, 14), REDOME has two features indicating that African ancestry is underrepresented, despite the registry’s large size. First, there are proportionally fewer individuals classified as “Mixed” in REDOME as compared to the census for Brazil. Second, individuals with greater African ancestry have decreased chances of finding a match. Increasing REDOME’s African ancestry component, as shown by our findings, is expected to increase the chance of individuals of African ancestry finding a match (**Figure 4**).

Once the underrepresented group has been identified, what is the best strategy for donor recruitment? It is economically unfeasible to determine the genetic ancestry of all donors. An alternative is to direct recruitment campaigns to regions/cities where the underrepresented group is more common, or to work with governmental and non-governmental organizations that represent these groups. The impact of a strategy directed to increase the presence of underrepresented groups in REDOME is likely to extend beyond Brazil, since between 2016-2018 REDOME exported 282 hematopoietic cells to Europe, the United States, and Asia (63).

### Caveats

In this study we resorted to imputation based on the SNP data to make HLA calls. The accuracy of imputation is reduced when the reference panel inadequately covers the diversity of the sample being imputed. Because the number of samples in available reference panels is still scarce, especially for admixed populations (64–67), alleles may have been incorrectly imputed. To evaluate the impact of imputation errors, we replicated our analyses for a subset of individuals with > 80% probability of correct imputation in all 5 HLA loci. In this dataset of HLA genotypes with a prediction of high accuracy, we find a greater proportion of individuals with at least one match (**Figure S17**, **Tables S11** and **S12**). This reflects the higher predicted accuracy of imputation for non-rare alleles and non-Africans, for which matching probabilities are also higher. However, the influence of ancestry on matching is highly concordant with the original conclusions, although some statistics are no longer significant (**Figure S17**, **Tables S11** and **S12**).

Our study was unable to explore the effects of Native American ancestry, which reaches 20% in Northern Brazil (39, 68). While a study of induced pluripotent stem cell (iPSC) banks in the USA showed similar match rates for Caucasians and Native Americans (48% and 46%, likely due to their low genetic diversities) (69), this remains to be explored in Brazil. However, the fact that most Native American alleles are present in REDOME (78% of endemic alleles were observed; https://genevol.ib.usp.br/wp-content/uploads/2020/05/AFND.html), and that their genetic diversity is very low, makes it likely that the finding of Pappas et al. (69) will also apply to Brazil, with match rates for Native Americans being similar to those of Europeans.

The nature of REDOME’s data provides further challenges to our analyses. The data results from samples collected over a span of 30 years, resulting in substantial heterogeneity in the resolution of typing, and the number of loci typed per individual (**Table S4**). The samples collected earlier are at low resolution, whereas the most recent samples have the medium and high-resolution (including Next Generation Sequence data). As a consequence, our results for low and medium resolution, and across different numbers of loci, involve different sets of individuals and sample sizes. Given our focus on the role of ancestry and self-identification, we investigated whether there are differences in the distribution of IBGE categories across the data at different levels of resolution (or typed at a different number of loci). We found that the differences are very small (**Figure S18**), and therefore should not affect our findings.

Over most of the time of REDOME’s operation, newly recruited voluntary donors were only typed for *HLA-A, -B* and *-DRB1*, with genotyping at *HLA-C* and *-DQB1*, as well as high resolution typing conditioned to an initial match at 6/6 (or 5/6). Throughout the paper, we analyze 6/6, 8/8 and 10/10 low resolution typing, despite the fact that successful transplantation requires full or partial matching at high resolution. Our results for medium resolution, 8/8 and 10/10 matching show that the effect of African ancestry becomes even stronger when additional loci are used. While the numbers presented cannot be used directly for clinical practice, the trends we identify are expected to hold in an analysis with high-resolution typing. We therefore believe that the true impact of ancestry on chances of finding a match may in fact be found to be even stronger than we report, when high resolution data for 5 loci become widely available in REDOME.

A final caveat refers to the fact that our study only queries the presence of a matching in REDOME, whereas the chance of finding a donor also involves the ability to contact the potential donors, their willingness to perform the transplant, confirmatory HLA typing at high resolution, and a medical evaluation of the donor’s health. When these factors were taken into account, Gragert et al. (2014) (8) found that in the US donor availability for “Black” patients was 23%, compared to 51% for “White” patients. Since we do not have all this information for the Brazilian sample, we were unable to address these effects. However, the results of Gragert et al. (2014) (8) indicate that donor availability will be lower than the match rates we report, and that the effect of African ancestry on the chances of finding a match may be even greater than we document.

### Conclusion

We have shown that among patients seeking HSCT, those with a higher African ancestry face greater difficulty in locating potential donors. Given that more than half of the Brazilian population self-identifies as “Mixed” or “Black” [45.4% and 9.1%; (40)], and that these categories on average have increased African ancestry, this implies a reduced access to HSCT for a large proportion of Brazilians. Difficulty in obtaining a donor for HSTC is one among other forms of inequality faced by “Black” or “Mixed” Brazilians, who also carry a higher burden with respect to infant mortality (70), maternal mortality, risk of stroke (71), and more recently in the proportion of deaths due to COVID-19 (72). In these cases, self-identification is mainly a predictor of mortality as a consequence of the association to socio-economic status (71). In the case of access to donors in REDOME, we show that African ancestry is correlated with decreased chances of finding a match as an outcome of higher population-level diversity among African populations, associated with an underrepresentation of Brazilians of African ancestry in REDOME.

REDOME has a substantially higher frequency of “White” (54.6%) than “Mixed” or “Black” individuals (23.4% and 7.2%, respectively), implying a marked deficit of non-white categories in relation to that of Brazil as a whole. To illustrate a way to address this deficit, consider that individuals with two chromosomes with an African MHC have more matches to “Blacks” than to “Whites” (27.4% vs. 26.6%), even though “Blacks” represent only 7.2% of REDOME. This indicates that an increase in the proportion of individuals with African ancestry within REDOME will decrease the inequality in access to HSCT. This can be achieved, for example, by directing recruitment of new voluntary donors to regions where African ancestry is greater.

## Data Availability Statement

Publicly available datasets were analyzed in this study. This data can be found here: The data reported in this paper have been deposited in the European Nucleotide Archive European Nucleotide Archive (PRJEB26388 (ERP108374)), under EPIGEN Committee Controlled Access mode. And in the dbGAP (phs001972.v1.p1).

## Ethics Statements

The studies involving human participants were reviewed and approved by Comissão Nacional de Ética em Pesquisa - Brazil (CONEP), the local ethics committees at each participating center, the Institutional Review Boards at the University of California, San Francisco, the REDS-III data coordinating center, RTI International, approved the study. The patients/participants provided their written informed consent to participate in this study.

## Author Contributions

KN, LP, and DM designed the study. KN, VA, MS, and AS analyzed the data. KN, VA, and DM wrote the manuscript. DO, CD, FK, ET-S, VR, AC-P, PL, MF-P, CM, SK, BC, BW, ES, and LP discussed, reviewed, and edited manuscript. All authors contributed to the article and approved the submitted version.

## Funding

This work was supported by the United States National Institutes of Health - NIH (R01 GM075091; KN, VA, DM, BW); National Institutes of Health, National Heart, Lung, and Blood Institute (HHSN268201100007I; REDS-III); Conselho Nacional de Desenvolvimento Científico e Tecnológico - CNPq and Departamento de Ciência e Tecnologia da Secretaria de Ciência, Tecnologia e Insumos Estratégicos do Ministério da Saúde - Decit/SCTIE/MS (ET-S); Brazil Health Ministry – Transplant National System (proc. 25000.210075/2012-70; LP). São Paulo Funding Agency - FAPESP (2012/09950-9 KN and 2012/18010-0 DM). Minas Gerais State Research Agency (FAPEMIG RED-00314-16 ET-S).

## Conflict of Interest

The authors declare that the research was conducted in the absence of any commercial or financial relationships that could be construed as a potential conflict of interest.

## References

[1] Champlin RE Gale RP Role of bone marrow transplantation in the treatment of hematologic malignancies and solid tumors: critical review of syngeneic, autologous, and allogeneic transplants. Cancer Treat Rep (1984) 68:145–61. 6362862

[2] Singh AK McGuirk JP Allogeneic Stem Cell Transplantation: A Historical and Scientific Overview. Cancer Res (2016) 76:6445–51. 10.1158/0008-5472.CAN-16-1311 27784742

[3] Tiercy J-M How to select the best available related or unrelated donor of hematopoietic stem cells? Haematologica (2016) 101:680–7. 10.3324/haematol.2015.141119 PMC5013969 27252513

[4] Duquesnoy RJ Witvliet M Doxiadis IIN de Fijter H Claas FHJ HLAMatchmaker-based strategy to identify acceptable HLA class I mismatches for highly sensitized kidney transplant candidates. Transpl Int (2004) 17:22–30. 10.1007/s00147-003-0641-z 12955350

[5] Tiercy J-M Unrelated hematopoietic stem cell donor matching probability and search algorithm. Bone Marrow Res (2012) 2012:695018. 10.1155/2012/695018 23198148 PMC3502776

[6] Ruggeri A Labopin M Savani B Paviglianiti A Blaise D Volt F Hematopoietic stem cell transplantation with unrelated cord blood or haploidentical donor grafts in adult patients with secondary acute myeloid leukemia, a comparative study from Eurocord and the ALWP EBMT. Bone Marrow Transplant (2019) 54:1987–94. 10.1038/s41409-019-0582-5 31150016

[7] Tiercy J-M Claas F Impact of HLA diversity on donor selection in organ and stem cell transplantation. Hum Hered (2013) 76:178–86. 10.1159/000358798 24861862

[8] Gragert L Eapen M Williams E Freeman J Spellman S Baitty R HLA match likelihoods for hematopoietic stem-cell grafts in the U.S. registry. N Engl J Med (2014) 371:339–48. 10.1056/NEJMsa1311707 PMC5965695 25054717

[9] Solberg OD Mack SJ Lancaster AK Single RM Tsai Y Sanchez-Mazas A Balancing selection and heterogeneity across the classical human leukocyte antigen loci: a meta-analytic review of 497 population studies. Hum Immunol (2008) 69:443–64. 10.1016/j.humimm.2008.05.001 PMC2632948 18638659

[10] Fernandez Vina MA Hollenbach JA Lyke KE Sztein MB Maiers M Klitz W Tracking human migrations by the analysis of the distribution of HLA alleles, lineages and haplotypes in closed and open populations. Philos Trans R Soc Lond B Biol Sci (2012) 367:820–9. 10.1098/rstb.2011.0320 PMC3267126 22312049

[11] Bergstrom TC Garratt R Sheehan-Connor D Stem Cell Donor Matching for Patients of Mixed Race. B E J Econom Anal Policy (2012) 12:746. 10.1515/1935-1682.3275

[12] Pidala J Kim J Schell M Lee SJ Hillgruber R Nye V Race/ethnicity affects the probability of finding an HLA-A, -B, -C and -DRB1 allele-matched unrelated donor and likelihood of subsequent transplant utilization. Bone Marrow Transplant (2013) 48:346–50. 10.1038/bmt.2012.150 PMC4500122 22863723

[13] Barker JN Boughan K Dahi PB Devlin SM Maloy MA Naputo K Racial disparities in access to HLA-matched unrelated donor transplants: a prospective 1312-patient analysis. Blood Adv (2019) 3:939–44. 10.1182/bloodadvances.2018028662 PMC6457223 30917950

[14] Bergstrom TC Garratt RJ Sheehan-Connor D One Chance in a Million: Altruism and the Bone Marrow Registry. Am Econ Rev (2009) 99:1309–34. 10.1257/aer.99.4.1309 29508972

[15] Rosenberg NA Kang JTL Genetic Diversity and Societally Important Disparities. Genetics (2015) 201:1–12. 10.1534/genetics.115.176750 26354973 PMC4566256

[16] Bryc K Durand EY Macpherson JM Reich D Mountain JL The genetic ancestry of African Americans, Latinos, and European Americans across the United States. Am J Hum Genet (2015) 96:37–53. 10.1016/j.ajhg.2014.11.010 25529636 PMC4289685

[17] Hollenbach JA Saperstein A Albrecht M Vierra-Green C Parham P Norman PJ Race, Ethnicity and Ancestry in Unrelated Transplant Matching for the National Marrow Donor Program: A Comparison of Multiple Forms of Self-Identification with Genetics. PLoS One (2015) 10:e0135960. 10.1371/journal.pone.0135960 26287376 PMC4545604

[18] Lima-Costa MF Rodrigues LC Barreto ML Gouveia M Horta BL Mambrini J Genomic ancestry and ethnoracial self-classification based on 5,871 community-dwelling Brazilians (The Epigen Initiative). Sci Rep (2015) 5:9812. 10.1038/srep09812 25913126 PMC5386196

[19] Sankar P Cho MK Genetics. Toward a new vocabulary of human genetic variation. Science (2002) 298:1337–8. 10.1126/science.1074447 PMC2271140 12434037

[20] Gomes MB Gabrielli AB Santos DC Pizarro MH Barros BSV Negrato CA Self-reported color-race and genomic ancestry in an admixed population: A contribution of a nationwide survey in patients with type 1 diabetes in Brazil. Diabetes Res Clin Pract (2018) 140:245–52. 10.1016/j.diabres.2018.03.021 29574106

[21] Santos DC Porto LC Oliveira RV Secco D Hanhoerderster L Pizarro MH HLA class II genotyping of admixed Brazilian patients with type 1 diabetes according to self-reported color/race in a nationwide study. Sci Rep (2020) 10:6628. 10.1038/s41598-020-63322-y 32313169 PMC7170860

[22] Carneiro-Proietti ABF Kelly S Miranda Teixeira C Sabino EC Alencar CS Capuani L Clinical and genetic ancestry profile of a large multi-centre sickle cell disease cohort in Brazil. Br J Haematol (2018) 182:895–908. 10.1111/bjh.15462 30027669 PMC8019534

[23] Kehdy FSG Gouveia MH Machado M Magalhães WCS Horimoto AR Horta BL Origin and dynamics of admixture in Brazilians and its effect on the pattern of deleterious mutations. Proc Natl Acad Sci U S A (2015) 112:8696–701. 10.1073/pnas.1504447112 PMC4507185 26124090

[24] Barreto ML Cunha SS Alcântara-Neves N Carvalho LP Cruz AA Stein RT Risk factors and immunological pathways for asthma and other allergic diseases in children: background and methodology of a longitudinal study in a large urban center in Northeastern Brazil (Salvador-SCAALA study). BMC Pulm Med (2006) 6:15. 10.1186/1471-2466-6-15 16796729 PMC1559717

[25] Lima-Costa MF Firmo JOA Uchoa E Cohort profile: the Bambui (Brazil) Cohort Study of Ageing. Int J Epidemiol (2011) 40:862–7. 10.1093/ije/dyq143 20805109

[26] Victora CG Barros FC Cohort profile: the 1982 Pelotas (Brazil) birth cohort study. Int J Epidemiol (2006) 35:237–42. 10.1093/ije/dyi290 16373375

[27] Kuniholm MH Xie X Anastos K Xue X Reimers L French AL Human leucocyte antigen class I and II imputation in a multiracial population. Int J Immunogenet (2016) 43:369–75. 10.1111/iji.12292 PMC5118156 27774761

[28] Karnes JH Shaffer CM Bastarache L Gaudieri S Glazer AM Steiner HE Comparison of HLA allelic imputation programs. PLoS One (2017) 12:e0172444. 10.1371/journal.pone.0172444 28207879 PMC5312875

[29] Pappas DJ Lizee A Paunic V Beutner KR Motyer A Vukcevic D Significant variation between SNP-based HLA imputations in diverse populations: the last mile is the hardest. Pharmacogenomics J (2018) 18:367–76. 10.1038/tpj.2017.7 PMC5656547 28440342

[30] Zheng X Shen J Cox C Wakefield JC Ehm MG Nelson MR HIBAG–HLA genotype imputation with attribute bagging. Pharmacogenomics J (2014) 14:192–200. 10.1038/tpj.2013.18 23712092 PMC3772955

[31] Gourraud P-A Khankhanian P Cereb N Yang SY Feolo M Maiers M HLA diversity in the 1000 genomes dataset. PLoS One (2014) 9:e97282. 10.1371/journal.pone.0097282 24988075 PMC4079705

[32] Abi-Rached L Gouret P Yeh J-H Di Cristofaro J Pontarotti P Picard C Immune diversity sheds light on missing variation in worldwide genetic diversity panels. PLoS One (2018) 13:e0206512. 10.1371/journal.pone.0206512 30365549 PMC6203392

[33] 1000 Genomes Project Consortium Auton A Brooks LD Durbin RM Garrison EP Kang HM A global reference for human genetic variation. Nature (2015) 526:68–74. 10.1038/nature15393 26432245 PMC4750478

[34] Borda V Alvim I Aquino MM Silva C Soares-Souza GB Leal TP The genetic structure and adaptation of Andean highlanders and Amazonian dwellers is influenced by the interplay between geography and culture. bioRxiv (2020) 174:1–17. 10.1101/2020.01.30.916270 PMC7768732 33277433

[35] Alexander DH Novembre J Lange K Fast model-based estimation of ancestry in unrelated individuals. Genome Res (2009) 19:1655–64. 10.1101/gr.094052.109 PMC2752134 19648217

[36] Maples BK Gravel S Kenny EE Bustamante CD RFMix: a discriminative modeling approach for rapid and robust local-ancestry inference. Am J Hum Genet (2013) 93:278–88. 10.1016/j.ajhg.2013.06.020 PMC3738819 23910464

[37] Delaneau O Marchini J Zagury J-F A linear complexity phasing method for thousands of genomes. Nat Methods (2011) 9:179–81. 10.1038/nmeth.1785 22138821

[38] IBGE Brasil: 500 anos de povoamento. Centro de Documentação e Disseminação de Informações (2007). Available at: https://biblioteca.ibge.gov.br/visualizacao/livros/liv6687.pdf (Accessed July 14, 2020).

[39] Pena SDJ Di Pietro G Fuchshuber-Moraes M Genro JP Hutz MH Kehdy F de SG The genomic ancestry of individuals from different geographical regions of Brazil is more uniform than expected. PLoS One (2011) 6:e17063. 10.1371/journal.pone.0017063 21359226 PMC3040205

[40] População | IBGE Available at: https://www.ibge.gov.br/estatisticas/sociais/populacao.html (Accessed July 14, 2020).

[41] Lins TC Vieira RG Abreu BS Gentil P Moreno-Lima R Oliveira RJ Genetic heterogeneity of self-reported ancestry groups in an admixed Brazilian population. J Epidemiol (2011) 21:240–5. 10.2188/jea.je20100164 PMC3899415 21498954

[42] Flor-Park MV Kelly S Preiss L Custer B Carneiro-Proietti ABF Araujo AS Identification and Characterization of Hematopoietic Stem Cell Transplant Candidates in a Sickle Cell Disease Cohort. Biol Blood Marrow Transplant (2019) 25:2103–9. 10.1016/j.bbmt.2019.06.013 31229639

[43] Cançado RD Jesus JA A doença falciforme no Brasil. Rev Bras Hematol Hemoter (2007) 29:204–6. 10.1590/S1516-84842007000300002

[44] Dehn J Buck K Maiers M Confer D Hartzman R Kollman C 8/8 and 10/10 high-resolution match rate for the be the match unrelated donor registry. Biol Blood Marrow Transplant (2015) 21:137–41. 10.1016/j.bbmt.2014.10.002 25307419

[45] Bergstrom TC Garratt R Sheehan-Connor D Stem Cell Donor Matching for Patients of Mixed Race. UC Santa Barbara: Department of Economics, UCSB (2009). Available at: https://escholarship.org/uc/item/22w466q9.

[46] Ramachandran S Deshpande O Roseman CC Rosenberg NA Feldman MW Cavalli-Sforza LL Support from the relationship of genetic and geographic distance in human populations for a serial founder effect originating in Africa. Proc Natl Acad Sci U.S.A. (2005) 102:15942–7. 10.1073/pnas.0507611102 PMC1276087 16243969

[47] Prugnolle F Manica A Balloux F Geography predicts neutral genetic diversity of human populations. Curr Biol (2005) 15:R159–60. 10.1016/j.cub.2005.02.038 PMC1800886 15753023

[48] Meyer D Single RM Mack SJ Erlich HA Thomson G Signatures of demographic history and natural selection in the human major histocompatibility complex Loci. Genetics (2006) 173:2121–42. 10.1534/genetics.105.052837 PMC1569707 16702436

[49] Buhler S Sanchez-Mazas A HLA DNA sequence variation among human populations: molecular signatures of demographic and selective events. PloS One (2011) 6:e14643. 10.1371/journal.pone.0014643 21408106 PMC3051395

[50] Gouveia MH Borda V Leal TP Moreira RG Bergen AW Kehdy FSG Origins, Admixture Dynamics, and Homogenization of the African Gene Pool in the Americas. Mol Biol Evol (2020) 37:1647–56. 10.1093/molbev/msaa033 PMC7253211 32128591

[51] Lopes RB Identificação de haplótipos HLA presentes em pacientes inscritos no REREME que não possuem doador compatível no REDOME. [dissertation/master’s thesis]. Rio de Janeiro (RJ): Universidade do Estado do Rio de Janeiro (2018).

[52] Oevermann L Sodani P Status quo of allogeneic stem cell transplantation for patients with sickle cell disease using matched unrelated donors. Hematol Oncol Stem Cell Ther (2020) 13:116–9. 10.1016/j.hemonc.2019.12.004 32202244

[53] Angelucci E Matthes-Martin S Baronciani D Bernaudin F Bonanomi S Cappellini MD Hematopoietic stem cell transplantation in thalassemia major and sickle cell disease: indications and management recommendations from an international expert panel. Haematologica (2014) 99:811–20. 10.3324/haematol.2013.099747 PMC4008115 24790059

[54] Shenoy S Eapen M Panepinto JA Logan BR Wu J Abraham A A trial of unrelated donor marrow transplantation for children with severe sickle cell disease. Blood (2016) 128:2561–7. 10.1182/blood-2016-05-715870 PMC5123194 27625358

[55] Gluckman E de la Fuente J Cappelli B Scigliuolo GM Volt F Tozatto-Maio K The role of HLA matching in unrelated donor hematopoietic stem cell transplantation for sickle cell disease in Europe. Bone Marrow Transplant (2020) 55:1946–54. 10.1038/s41409-020-0847-z 32157246

[56] Pui C-H Evans WE Treatment of acute lymphoblastic leukemia. N Engl J Med (2006) 354:166–78. 10.1056/NEJMra052603 16407512

[57] Shenoy S Hematopoietic stem-cell transplantation for sickle cell disease: current evidence and opinions. Ther Adv Hematol (2013) 4:335–44. 10.1177/2040620713483063 PMC3766347 24082994

[58] Madbouly A Wang T Haagenson M Paunic V Vierra-Green C Fleischhauer K Investigating the Association of Genetic Admixture and Donor/Recipient Genetic Disparity with Transplant Outcomes. Biol Blood Marrow Transplant (2017) 23:1029–37. 10.1016/j.bbmt.2017.02.019 PMC5541944 28263917

[59] Sonnenberg FA Eckman MH Pauker SG Bone marrow donor registries: the relation between registry size and probability of finding complete and partial matches. Blood (1989) 74:2569–78. 2804379

[60] Schmidt AH Sauter J Pingel J Ehninger G Toward an optimal global stem cell donor recruitment strategy. PloS One (2014) 9:e86605. 10.1371/journal.pone.0086605 24497958 PMC3907384

[61] Maiers M Halagan M Joshi S Ballal HS Jagannatthan L Damodar S HLA match likelihoods for Indian patients seeking unrelated donor transplantation grafts: a population-based study. Lancet Haematol (2014) 1:e57–63. 10.1016/S2352-3026(14)70021-3 27030155

[62] Kwok J Guo M Yang W Ip P Chan GCF Ho J Estimation of optimal donor number in Bone Marrow Donor Registry: Hong Kong’s experience. Hum Immunol (2017) 78:610–3. 10.1016/j.humimm.2017.08.007 28865670

[63] Arrieta-Bolaños E Oliveira DC Barquera R Differential admixture, human leukocyte antigen diversity, and hematopoietic cell transplantation in Latin America: challenges and opportunities. Bone Marrow Transplant (2020) 55:496–504. 10.1038/s41409-019-0737-4 31695172

[64] Levin AM Adrianto I Datta I Iannuzzi MC Trudeau S McKeigue P Performance of HLA allele prediction methods in African Americans for class II genes HLA-DRB1, -DQB1, and -DPB1. BMC Genet (2014) 15:72. 10.1186/1471-2156-15-72 24935557 PMC4074844

[65] Nunes K Piovezan B Torres MA Pontes GN Kimura L Carnavalli JEP Population variation of HLA genes in rural communities in Brazil, the Quilombos from the Vale do Ribeira, São Paulo - Brazil. Hum Immunol (2016) 77:447–8. 10.1016/j.humimm.2016.04.007 27060779

[66] Meyer D Nunes K HLA imputation, what is it good for? Hum Immunol (2017) 78:239–41. 10.1016/j.humimm.2017.02.007 28317600

[67] Degenhardt F Wendorff M Wittig M Ellinghaus E Datta LW Schembri J Construction and benchmarking of a multi-ethnic reference panel for the imputation of HLA class I and II alleles. Hum Mol Genet (2019) 28:2078–92. 10.1093/hmg/ddy443 PMC6548229 30590525

[68] de Souza AM Resende SS de Sousa TN de Brito CFA A systematic scoping review of the genetic ancestry of the Brazilian population. Genet Mol Biol (2019) 42:495–508. 10.1590/1678-4685-GMB-2018-0076 31188926 PMC6905439

[69] Pappas DJ Gourraud P-A Le Gall C Laurent J Trounson A DeWitt N Proceedings: human leukocyte antigen haplo-homozygous induced pluripotent stem cell haplobank modeled after the california population: evaluating matching in a multiethnic and admixed population. Stem Cells Transl Med (2015) 4:413–8. 10.5966/sctm.2015-0052 PMC4414226 25926330

[70] Matijasevich A Victora CG Barros AJD Santos IS Marco PL Albernaz EP Widening ethnic disparities in infant mortality in southern Brazil: comparison of 3 birth cohorts. Am J Public Health (2008) 98:692–68. 10.2105/AJPH.2006.093492 PMC2376998 17761568

[71] Chor D Lima CR de A [Epidemiologic aspects of racial inequalities in health in Brazil]. Cad Saude Publica (2005) 21:1586–94. 10.1590/s0102-311x2005000500033 16158166

[72] BOLETIM EPIDEMIOLÓGICO ESPECIAL Doença pelo Coronavírus COVID-19. Ministério da Saúde (2020). Available at: http://saude.gov.br/images/pdf/2020/July/15/Boletim-epidemiologico-COVID-22.pdf (Accessed July 17, 2020).

