# Supplementary material for: How Ancestry Influences the Chances of Finding Unrelated Donors: An Investigation in Admixed Brazilians
Source: Front Immunol. 2020 Nov 6;11:584950. doi: 10.3389/fimmu.2020.584950 (PMC7677137; doi:10.3389/fimmu.2020.584950)
Supplement: Supplementary file 1 [file DataSheet_1.pdf]

Supplementary Material

How ancestry influences the chances of finding unrelated donors: an investigation in admixed Brazilians

Kelly Nunes, Vitor RC Aguiar, Márcio Silva, Alexandre C Sena, Danielli CM de Oliveira, Carla L Dinardo, Fernanda SG Kehdy, Eduardo Tarazona-Santos, Vanderson G Rocha, Anna Barbara F Carneiro-Proietti, Paula Loureiro, Miriam V Flor-Park, Claudia Maximo, Shannon Kelly, Brian Custer, Bruce S Weir, Ester C Sabino, Luís Cristóvão Porto, Diogo Meyer

1. Supplementary Figures

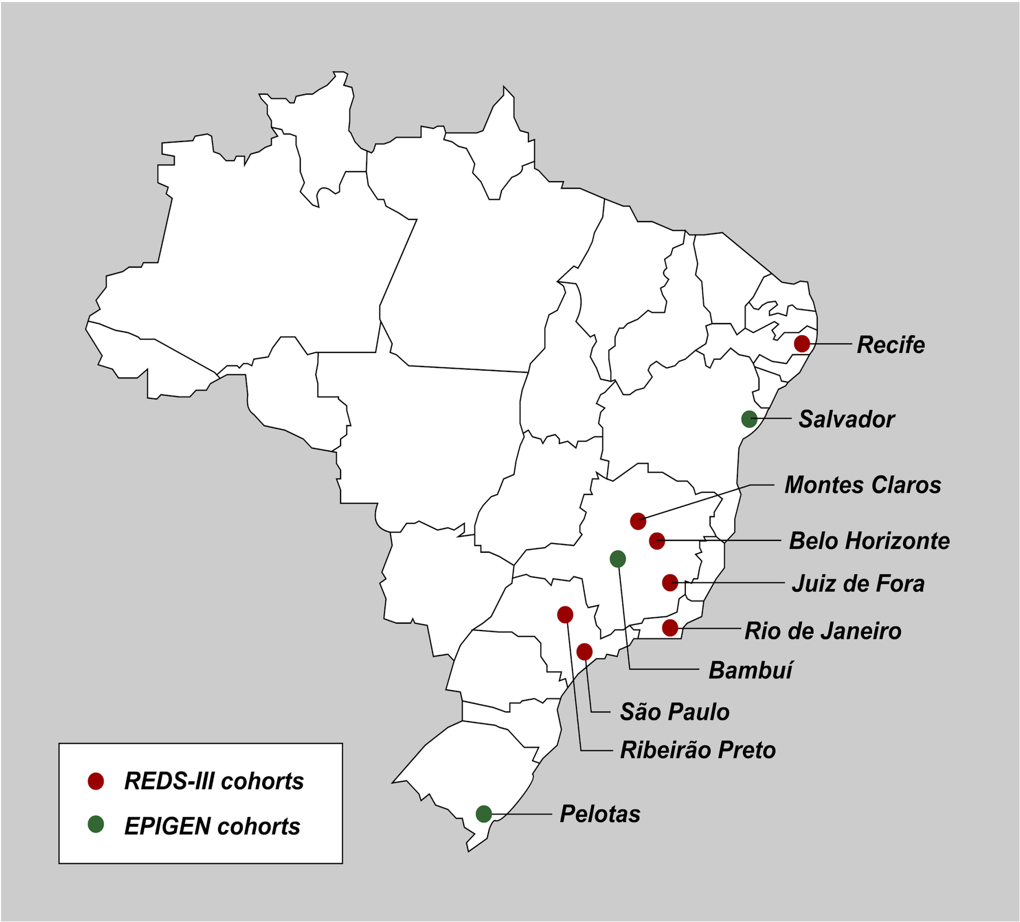

Figure S1. Geographic locations of the REDS-III and EPIGEN cohorts which compose the merged dataset used in our study.

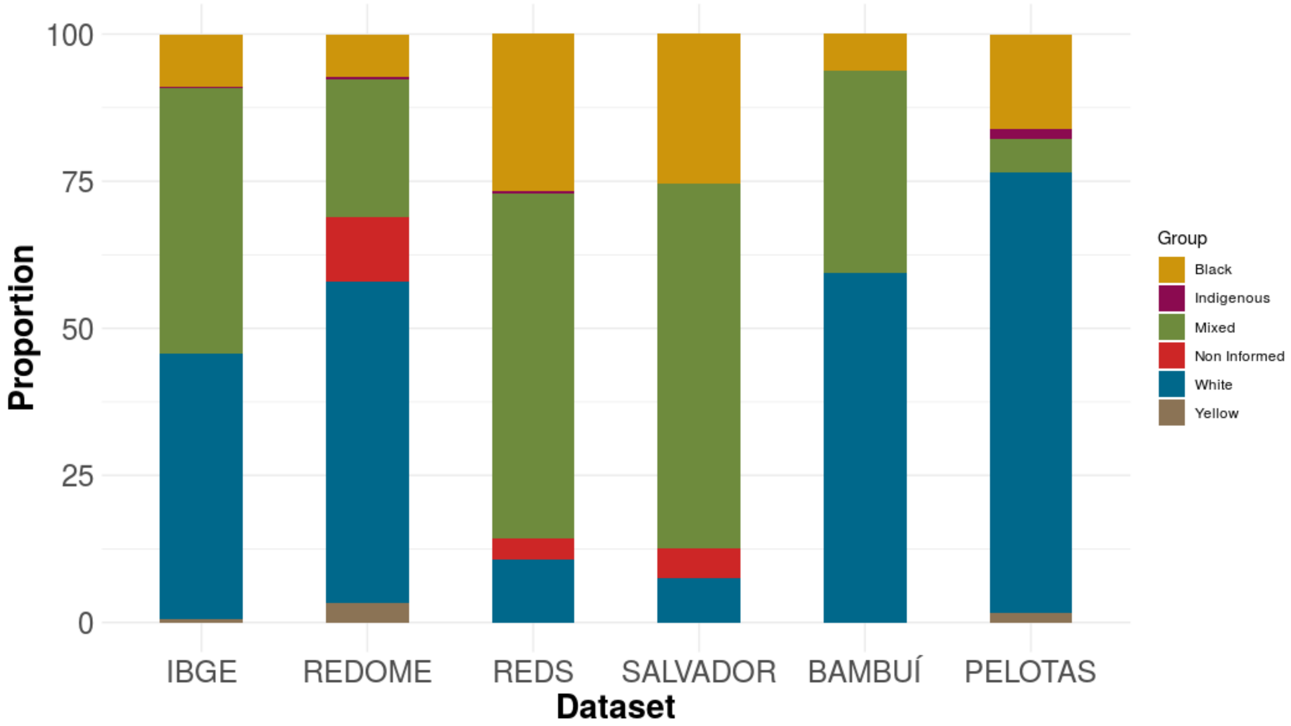

Figure S2. Self-Identification in the Brazilian population (IBGE) and in our datasets. Proportion of self-identified categories "Black", "Indigenous", "Mixed", "White", "Yellow" and "Non-Informed" for IBGE. REDOME, REDS-III and EPIGEN (Salvador, Bambuí and Pelotas).

Supplementary Material

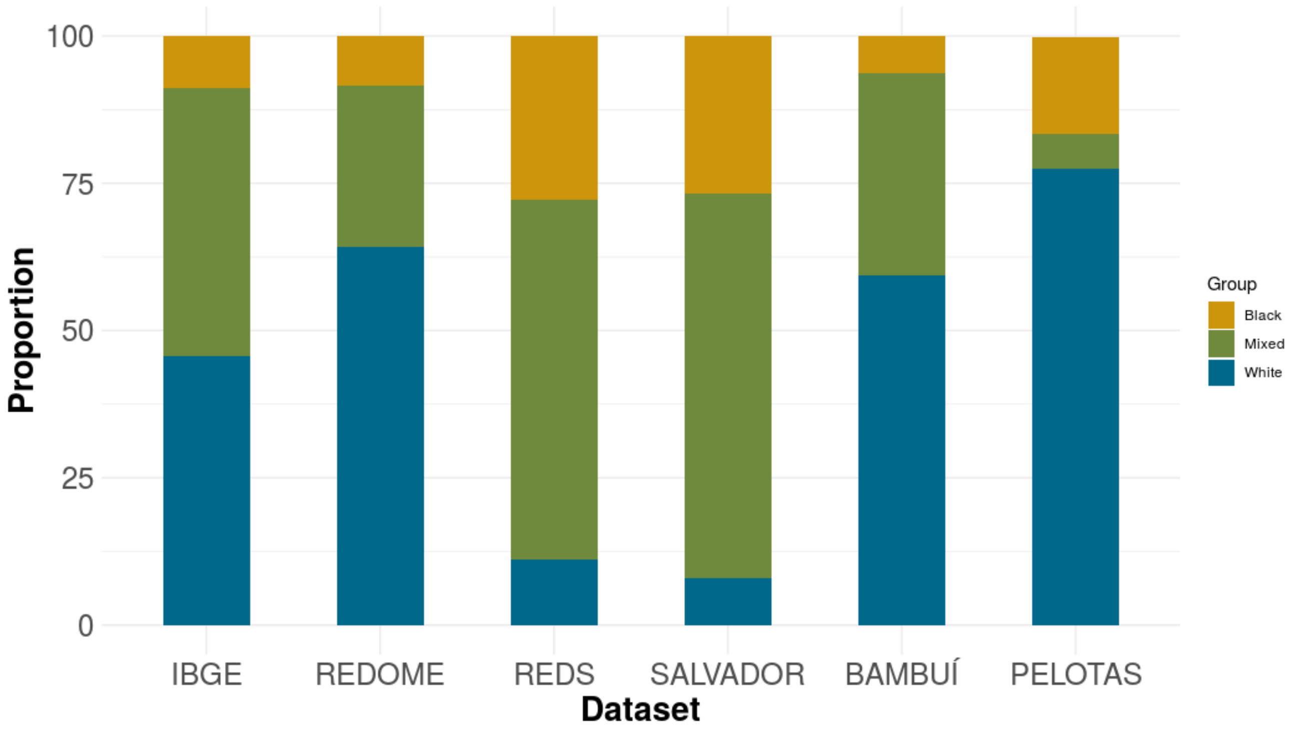

Figure S3. Self-Identification in the Brazilian population (IBGE) and in our datasets. Proportion of self-identified categories "Black", "Mixed" and "White" for IBGE. REDOME, REDS-III and EPIGEN (Bambuí, Salvador and Pelotas).

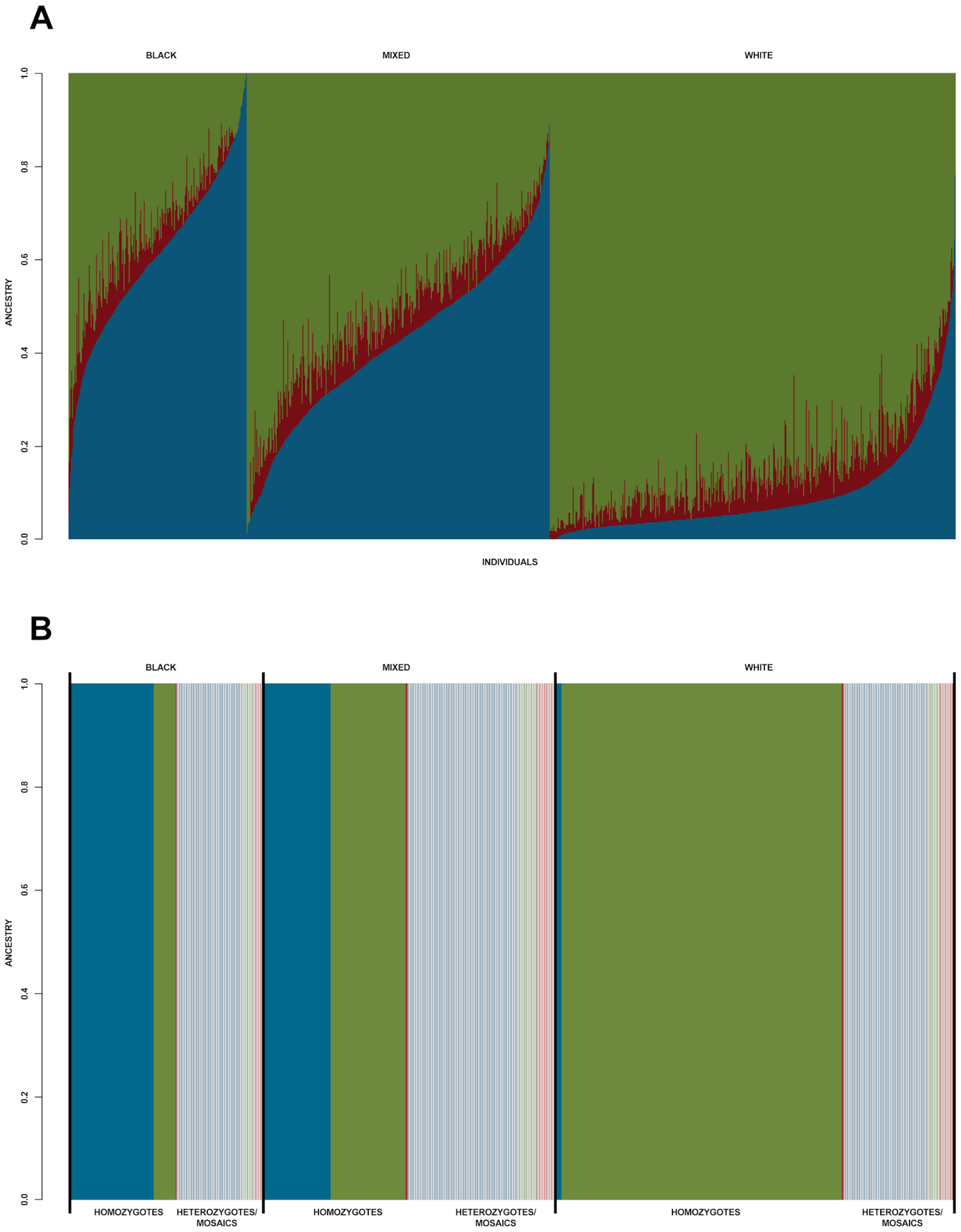

Figure S4. Ancestry inference. **A.** Self-identification and the proportion of genome wide ancestry for REDS+EPIGEN dataset samples. The vertical lines represent the proportion of ancestry inferred in each individual. The colors blue, green and red correspond to African, European and Native American ancestry respectively. **B.** Self-identification and the MHC ancestry for REDS+EPIGEN dataset samples. The vertical lines represent the proportion of ancestry inferred in each chromosome for the MHC region (each two lines represent one individual). The colors blue, green and red correspond to African. European and Native American ancestry respectively, Homozygotes correspond to a single ancestry in the MHC; heterozygotes refer to different ancestry between chromosome and mosaic to situations where there are more than one ancestry on the chromosome.

Supplementary Material

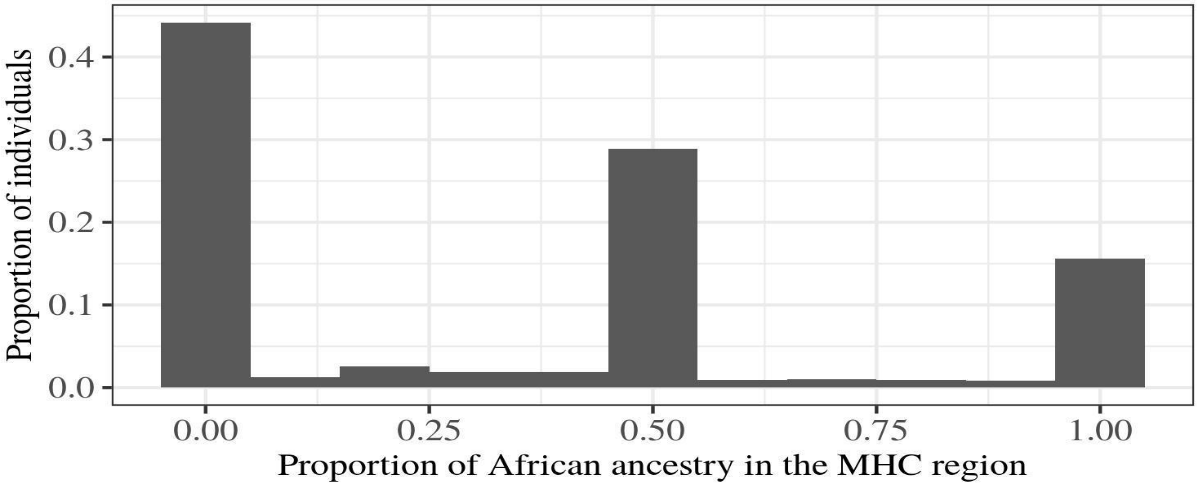

Figure S5. Histogram of the proportions of African ancestry in the MHC region. Proportions equal to 0. 0.5 and 1 correspond to 0, 1 and 2 chromosomes which are of African ancestry at the MHC, respectively. Intermediate values correspond to mosaics of different ancestries at the MHC.

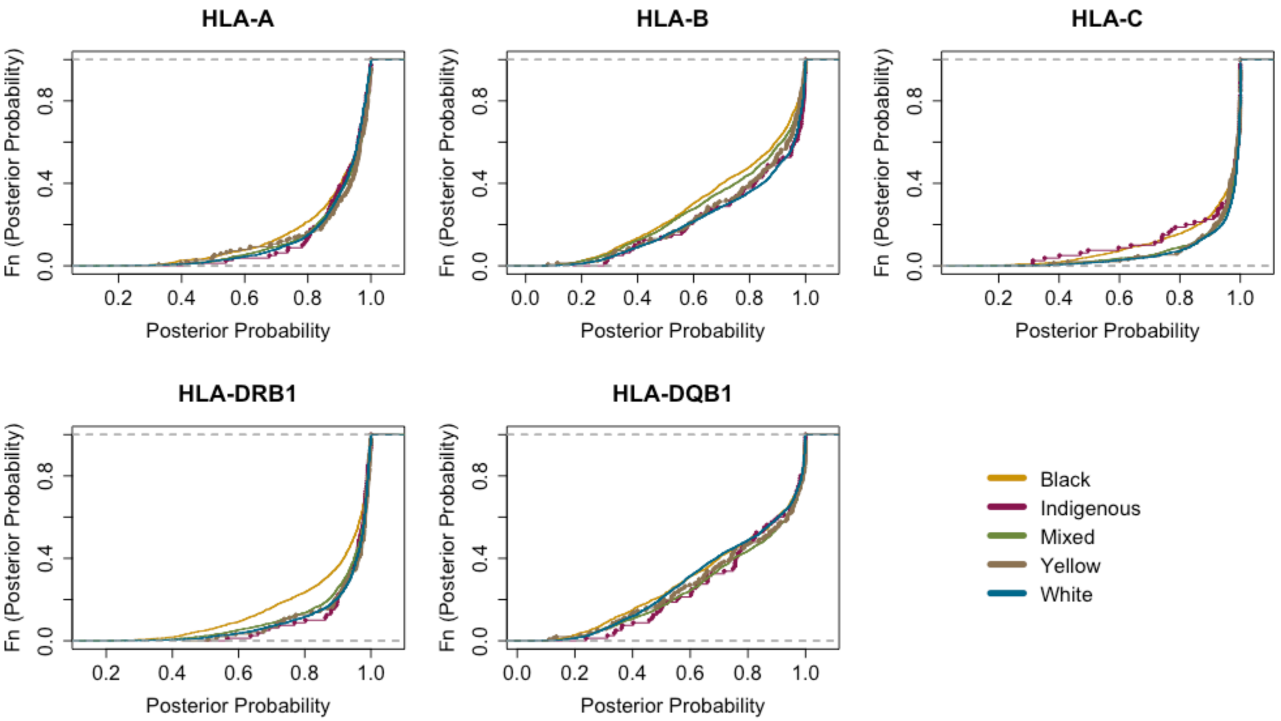

Figure S6. Empirical cumulative distribution function (ECDF) of HLA genotype (two-fields resolution) posterior probability for self-identification: Black (orange), Indigenous (purple), Mixed (green), Yellow (brown), White (blue). The ECDF organizes the posterior probabilities of the HLA genotypes, allowing the cumulative proportion of the genotypes to be observed throughout their distribution. In this case, we observed that self-declared individuals "Black", have lower posterior probabilities distribution than the other categories.

Supplementary Material

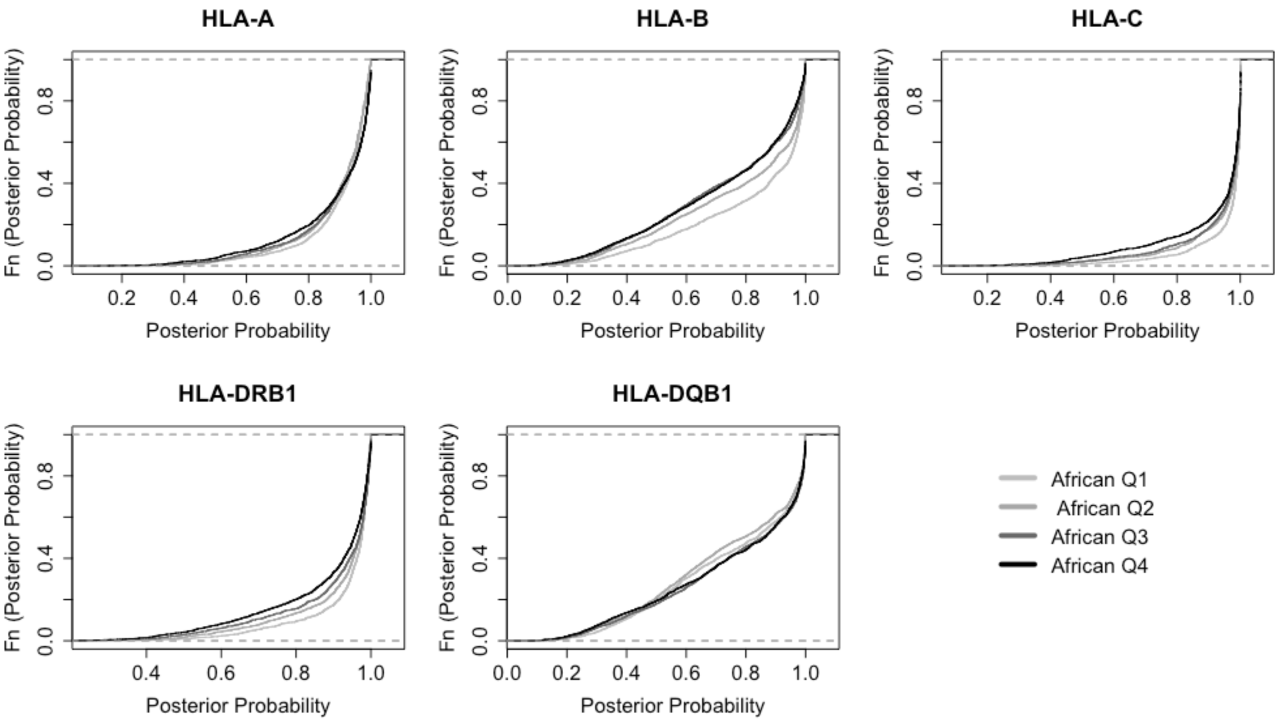

Figure S7. Empirical cumulative distribution function (ECDF) of HLA genotype (two-fields resolution) posterior probability for quartiles of African ancestry: Black (orange), Indigenous (purple), Mixed (green), Yellow (brown), White (blue). The ECDF organizes the posterior probabilities of the HLA genotypes, allowing the cumulative proportion of the genotypes to be observed throughout their distribution.

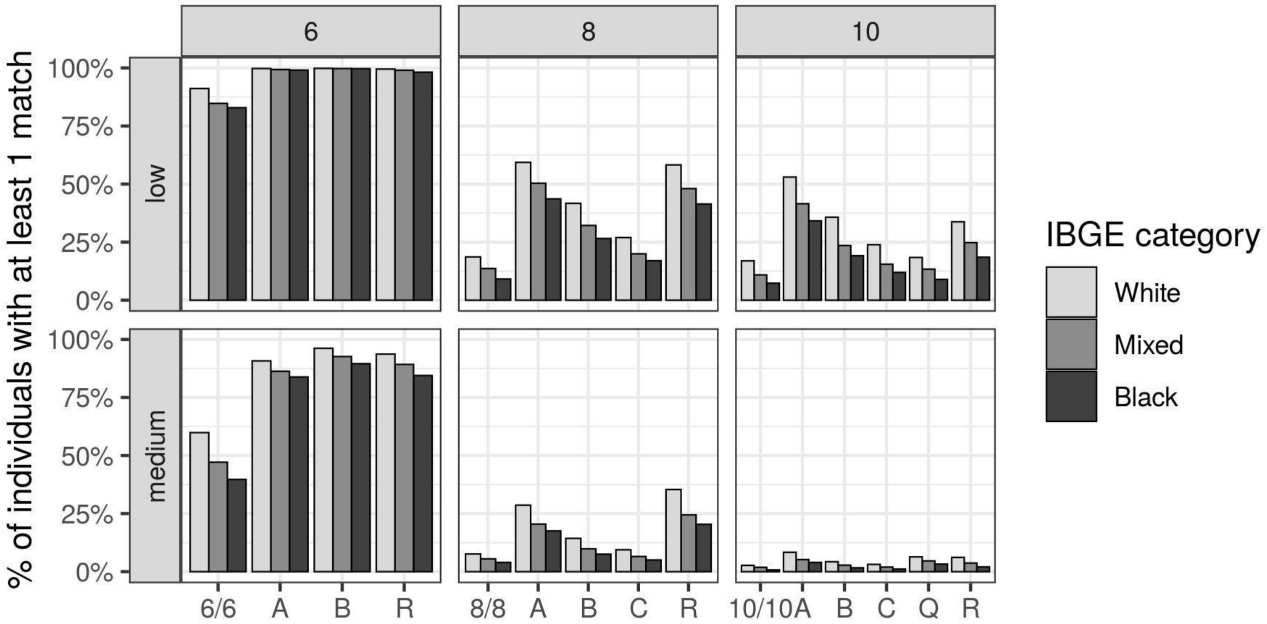

Figure S8. Percentage of individuals from different IBGE categories which found at least one match in REDOME. Columns correspond to matches at 6, 8, 10 alleles. Rows correspond to low and medium HLA typing resolution. The x-axis labels refer to either full matches (6/6, 8/8, 10/10) or the locus where a mismatch was allowed, A: *HLA-A*; B: *HLA-B*; C: *HLA-C*; Q: *HLA-DQB1*; R: *HLA-DRB1*.

Supplementary Material

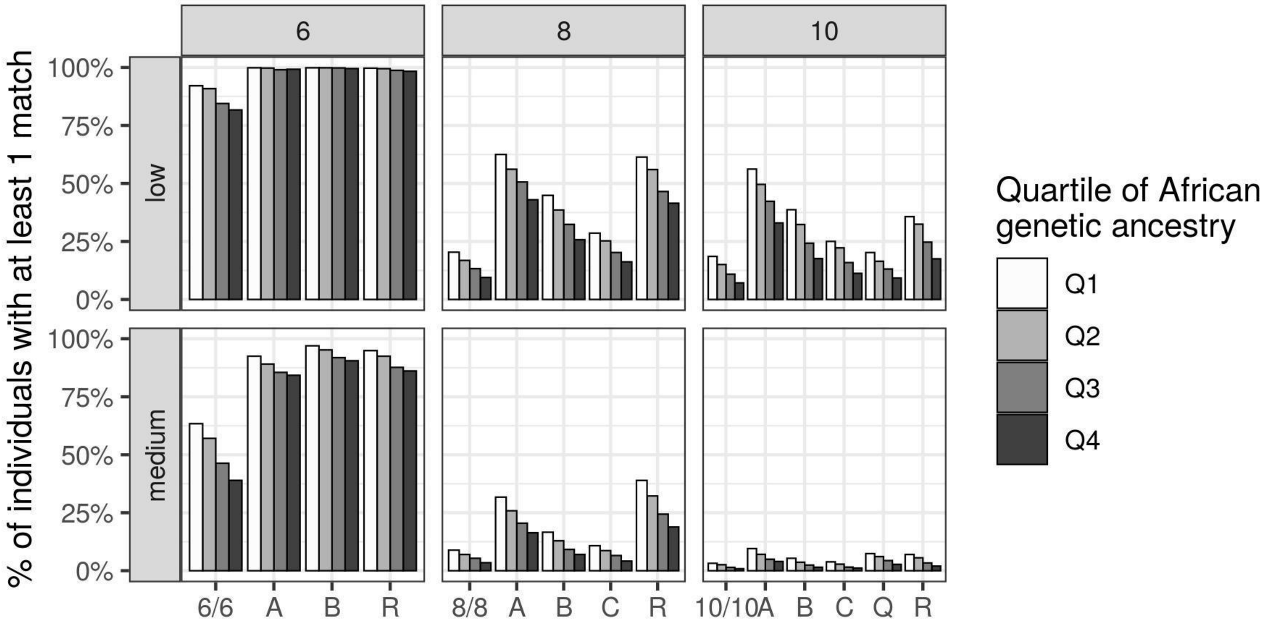

Figure S9. Percentage of individuals at different quartiles of African genetic ancestry which found at least one match in REDOME. Columns correspond to matches at 6, 8, 10 alleles. Rows correspond to low and medium HLA typing resolution. The x-axis labels refer to either full matches (6/6, 8/8, 10/10) or the locus where a mismatch was allowed, A: *HLA-A*; B: *HLA-B*; C: *HLA-C*; Q: *HLA-DQB1*; R: *HLA-DRB1*.

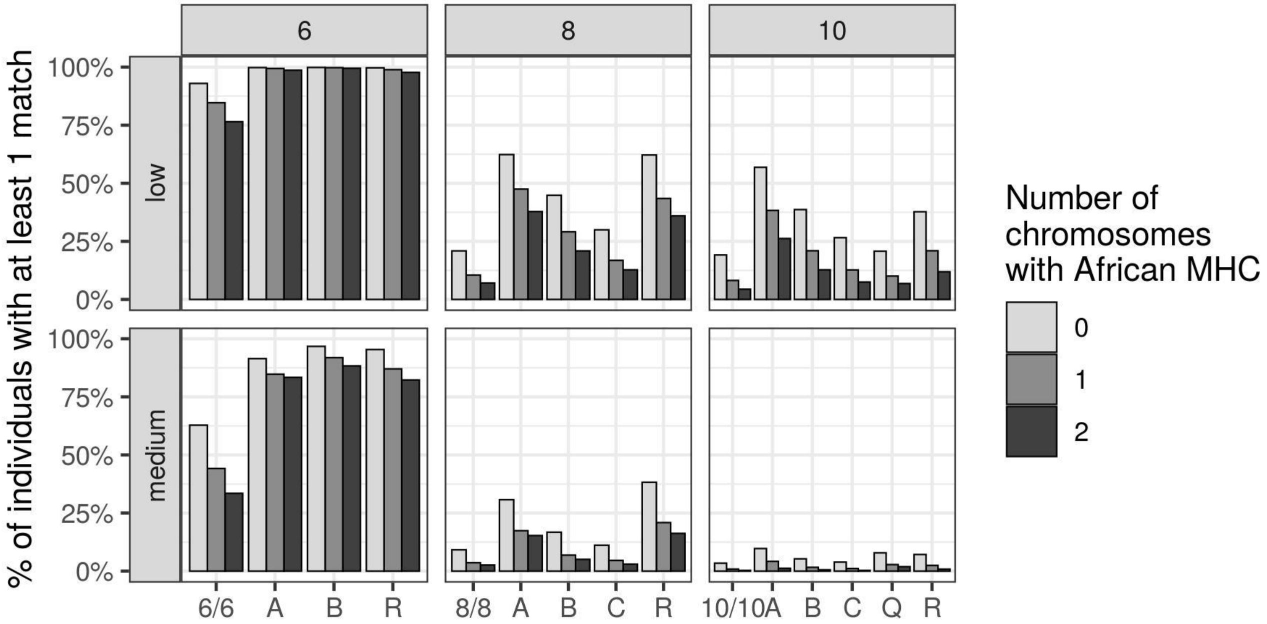

Figure S10. Percentage of individuals who found at least one match in REDOME. according to the number of chromosomes with African MHC which they carry. Columns correspond to matches at 6. 8. 10 alleles. Rows correspond to low and medium HLA typing resolution. The x-axis labels refer to either full matches (6/6, 8/8, 10/10) or the locus where a mismatch was allowed. A: *HLA-A*; B: *HLA-B*; C: *HLA-C*; Q: *HLA-DQB1*; R: *HLA-DRB1*.

Supplementary Material

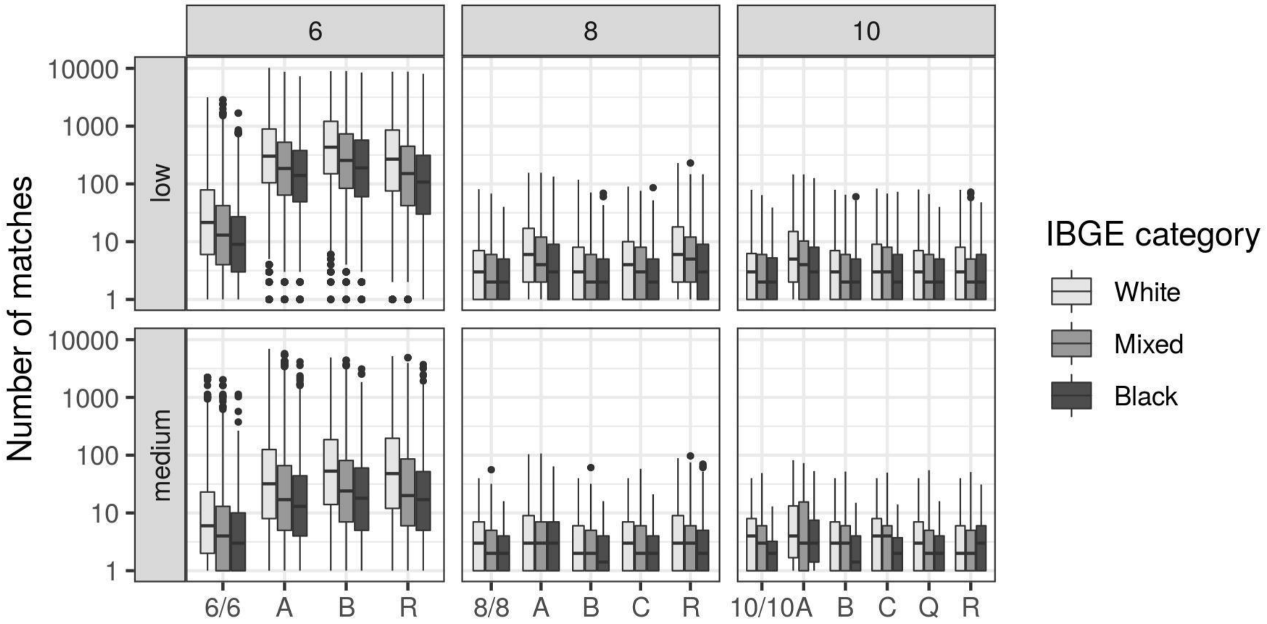

Figure S11. Boxplot of the number of matches in REDOME for individuals from different IBGE categories. Columns correspond to matches at 6, 8, 10 alleles. Rows correspond to low and medium HLA typing resolution. The x-axis labels refer to either full matches (6/6. 8/8. 10/10) or the locus where a mismatch was allowed. A: *HLA-A*; B: *HLA-B*; C: *HLA-C*; Q: *HLA-DQB1*; R: *HLA-DRB1*.

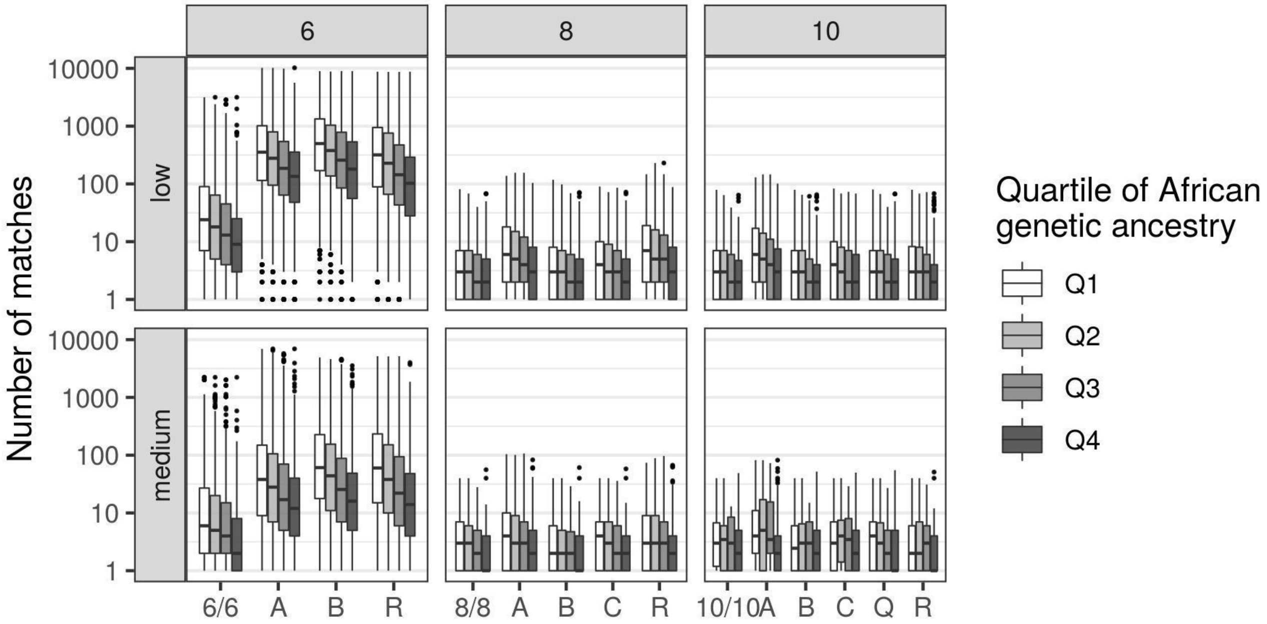

Figure S12. Boxplot of the number of matches in REDOME for individuals at different quartiles of African genetic ancestry. Columns correspond to matches at 6. 8. 10 alleles. Rows correspond to low and medium HLA typing resolution. The x-axis labels refer to either full matches (6/6, 8/8, 10/10) or the locus where a mismatch was allowed. A: *HLA-A*; B: *HLA-B*; C: *HLA-C*; Q: *HLA-DQB1*; R: *HLA-DRB1*.

Supplementary Material

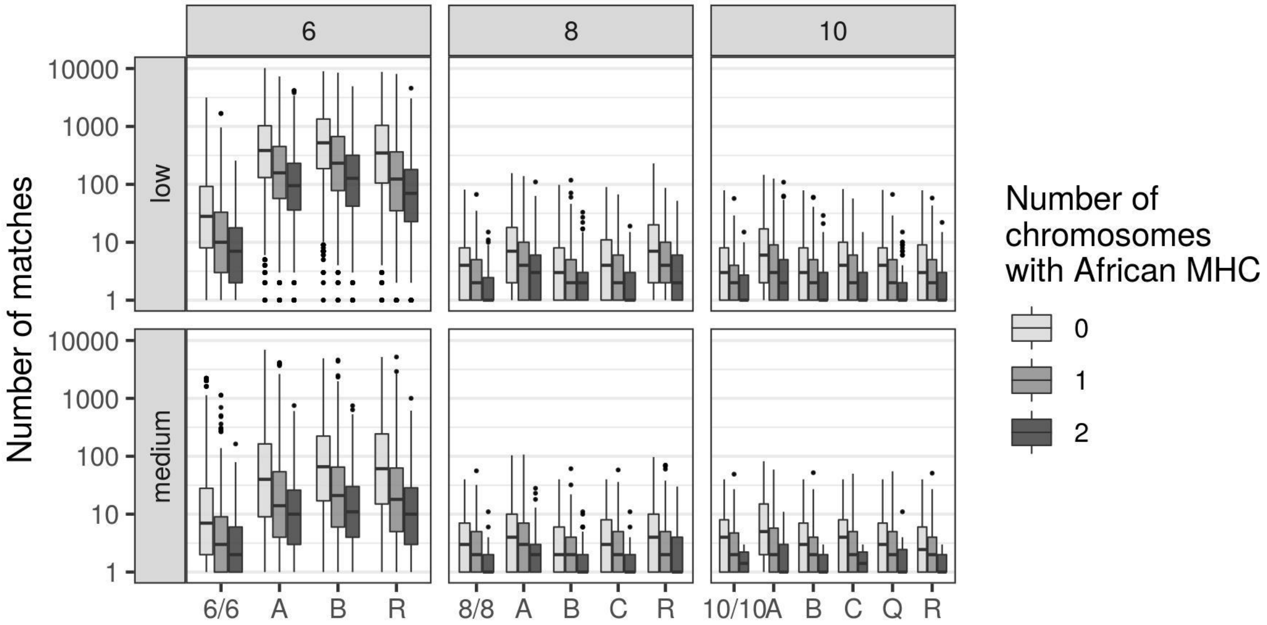

Figure S13. Boxplot of the number of matches in REDOME for individuals with a different number of chromosomes with African MHC. Columns correspond to matches at 6, 8, 10 alleles. Rows correspond to low and medium HLA typing resolution. The x-axis labels refer to either full matches (6/6, 8/8, 10/10) or the locus where a mismatch was allowed. A: *HLA-A*; B: *HLA-B*; C: *HLA-C*; Q: *HLA-DQB1*; R: *HLA-DRB1*.

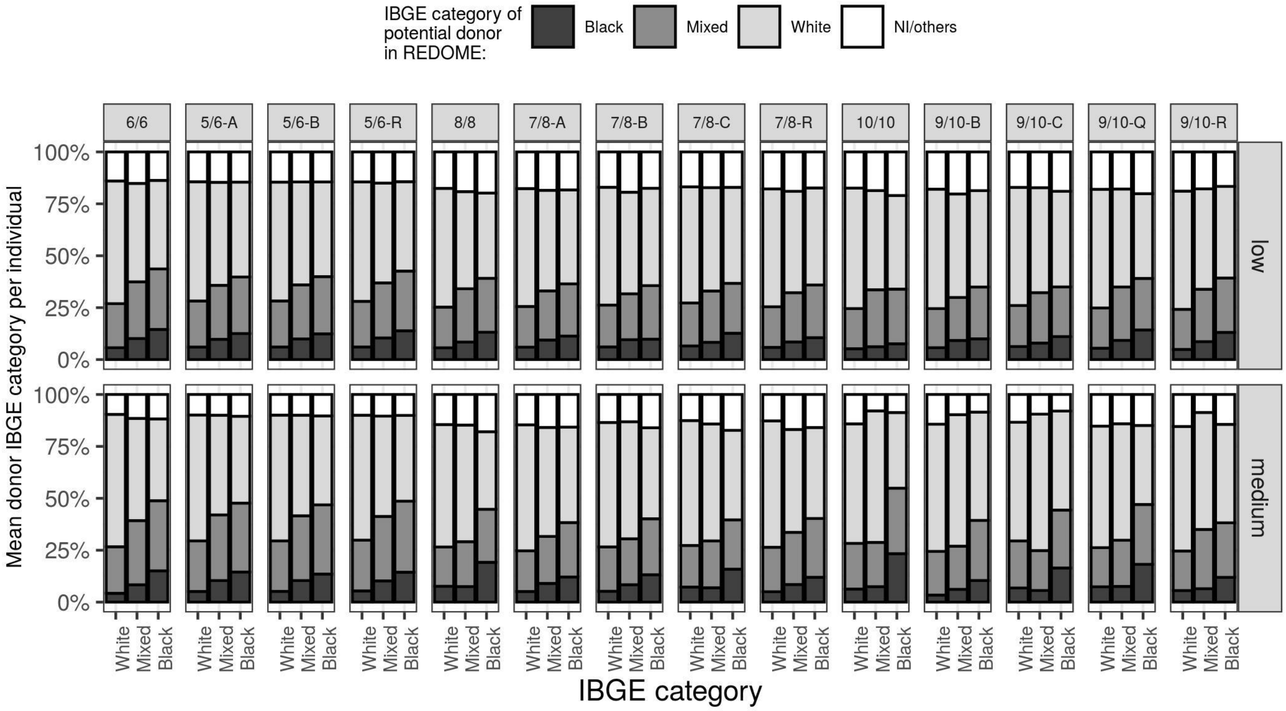

Figure S14. Mean IBGE category of donor in REDOME for individuals from different IBGE categories in the x-axis. Columns correspond to the number of alleles with full or partial match. For example, “6/6” refers to a full match at *HLA-A*, *HLA-B*, and *HLA-DRB1*, whereas “5/6-A” refers to a mismatch at *HLA-A*. Rows correspond to low and medium HLA typing resolution.

Supplementary Material

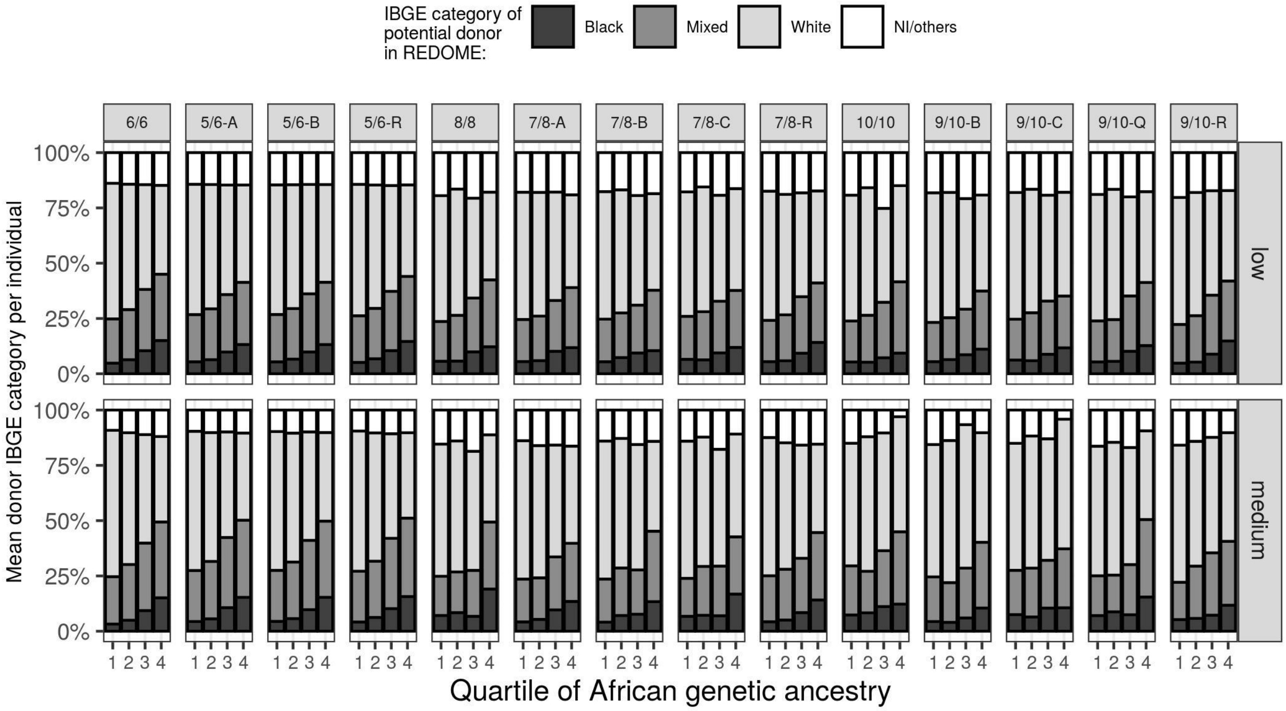

Figure S15. Mean IBGE category of donor in REDOME for individuals at different quartiles of African genetic ancestry. Columns correspond to the number of alleles with full or partial match. For example, "6/6" refers to a full match at *HLA-A*, *HLA-B*, and *HLA-DRB1*, whereas "5/6-A" refers to a mismatch at *HLA-A*. Rows correspond to low and medium HLA typing resolution.

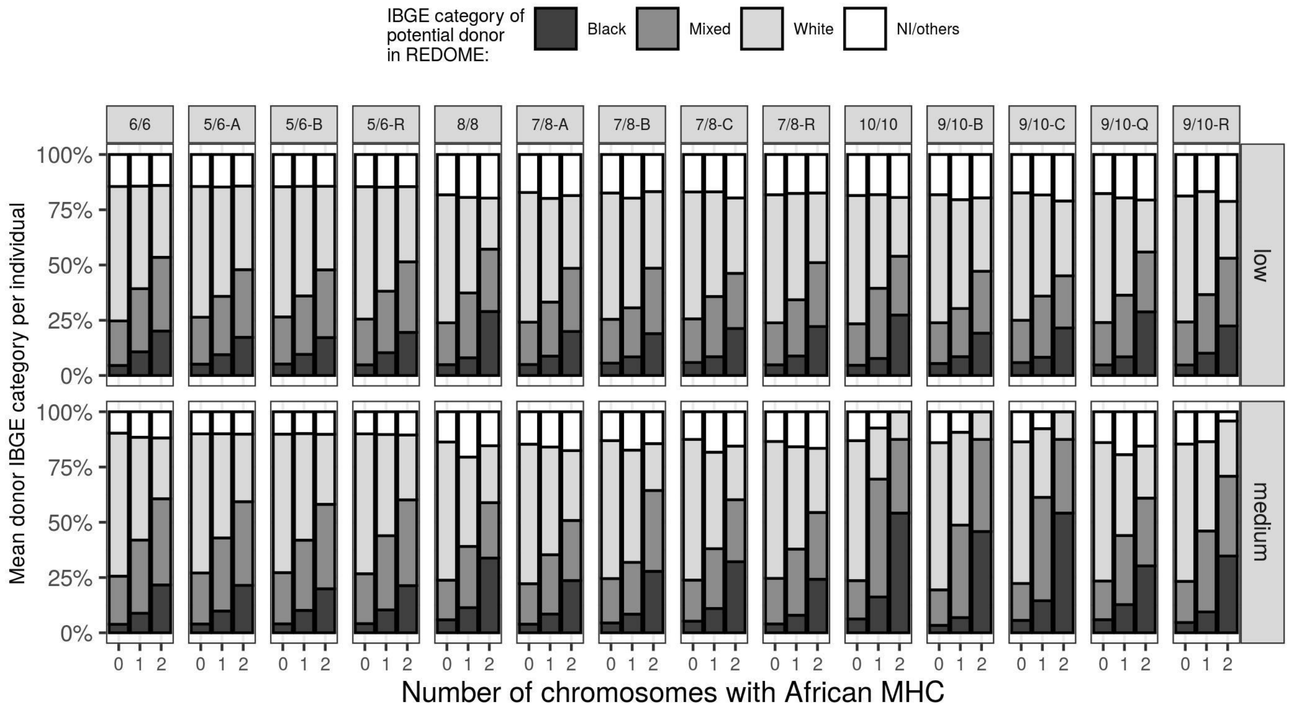

Figure S16. Mean IBGE category of donor in REDOME for individuals with a different number of chromosomes with African MHC. Columns correspond to the number of alleles with full or partial match. For example. "6/6" refers to a full match at *HLA-A*, *HLA-B*, and *HLA-DRB1*, whereas "5/6-A" refers to a mismatch at *HLA-A*. Rows correspond to low and medium HLA typing resolution.

Supplementary Material

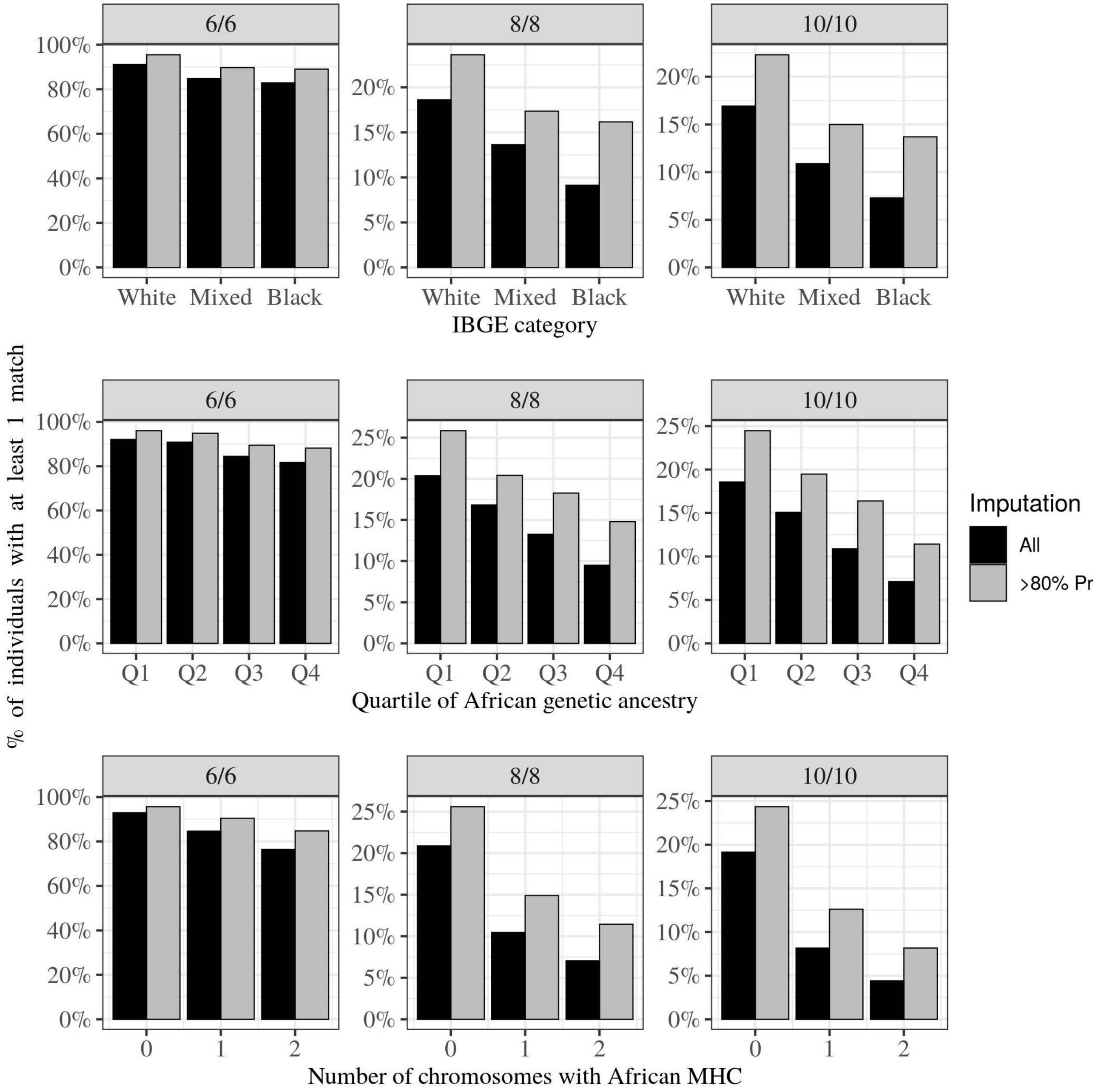

Figure S17. Percentage of individuals with at least one match in REDOME for low resolution 6/6, 8/8 and 10/10, in the whole merged dataset ("All") and in the subset of individuals with posterior probability of imputation >80% (">80% Pr"). Top row: IBGE categories; Middle row: Quartile of African ancestry; Bottom row: Number of chromosomes with African MHC per individual.

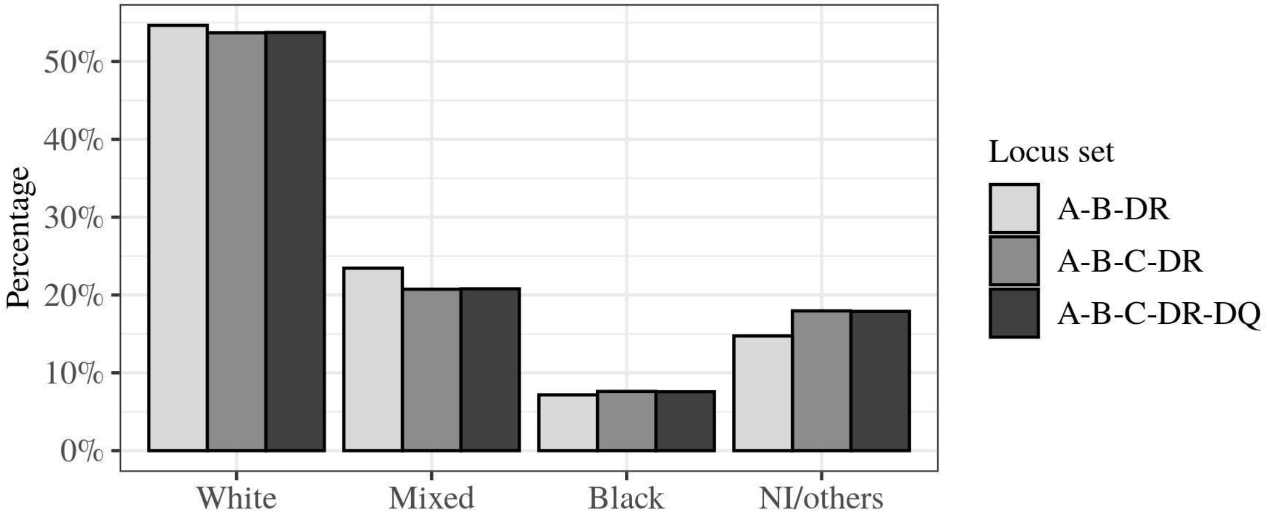

Figure S18. Percentage of "White", "Mixed", and "Black" individuals in the REDOME database according to the number of genotyped loci. NI/others: Non-informed category and other groups which are a minority in the database.

Supplementary Material

Supplementary Tables

Table S1. Out of bagging accuracy for each HLA imputation model (2 fields resolution).

GENE	N individuals reference panel	N HLA alleles reference panel	Average out-of-bag accuracy (sd) – TM array	Average out-of-bag accuracy (sd) - HumanOmin 2.5 array
HLA-A	2.503	82	95.37% ± 0.50%	95.62% ± 0.46%
HLA-B	2.498	154	90.97% ± 0.84%	89.60% ± 0.66%
HLA-C	2.503	62	98.28% ± 0.30%	97.88% ± 0.32%
HLA-DRB1	2.502	72	92.29% ± 0.75%	89.80% ± 0.67%
HLA-DQB1	2.502	30	97.88% ± 0.33%	98.22% ± 0.31%

Supplementary Material

Table S2. Summary statistics based on imputation posterior probabilities (2-fields resolution) by self-identifying group.

**Self-identified**	**Black**	**Indigenous**	**Mixed**	**Yellow**	**White**	**Non-Informed**
			HLA-A			
Min.	0.16	0.42	0.13	0.33	0.15	0.26
1st Qu.	0.83	0.86	0.86	0.88	0.86	0.72
Median	0.94	0.95	0.95	0.96	0.94	0.90
Mean	0.88	0.90	0.90	0.90	0.90	0.83
3rd Qu.	0.98	0.98	0.99	0.98	0.98	0.97
Max.	1.00	1.00	1.00	1.00	1.00	1.00
			HLA-B			
Min.	0.05	0.28	0.06	0.08	0.06	0.21
1st Qu.	0.55	0.65	0.56	0.64	0.65	0.54
Median	0.82	0.90	0.85	0.89	0.91	0.84
Mean	0.74	0.80	0.75	0.79	0.80	0.74
3rd Qu.	0.96	0.99	0.97	0.98	0.98	0.96
Max.	1.00	1.00	1.00	1.00	1.00	1.00
			HLA-C			
Min.	0.12	0.31	0.22	0.37	0.16	0.33
1st Qu.	0.91	0.92	0.95	0.94	0.96	0.75
Median	0.98	0.98	0.99	0.99	0.99	0.97
Mean	0.91	0.91	0.94	0.94	0.95	0.87
3rd Qu.	1.00	1.00	1.00	1.00	1.00	1.00
Max.	1.00	1.00	1.00	1.00	1.00	1.00
			HLA-DRB1			
Min.	0.20	0.57	0.14	0.51	0.21	0.50
1st Qu.	0.82	0.92	0.90	0.92	0.92	0.81
Median	0.95	0.97	0.97	0.98	0.97	0.93
Mean	0.87	0.93	0.91	0.93	0.92	0.87
3rd Qu.	0.99	0.99	0.99	0.99	0.99	0.98
Max.	1.00	1.00	1.00	1.00	1.00	1.00
			HLA-DQB1			
Min.	0.08	0.24	0.10	0.11	0.06	0.21
1st Qu.	0.54	0.62	0.61	0.58	0.54	0.54
Median	0.81	0.82	0.87	0.86	0.81	0.84
Mean	0.73	0.78	0.77	0.76	0.74	0.73
3rd Qu.	0.98	0.98	0.98	0.98	0.98	0.96
Max.	1.00	1.00	1.00	1.00	1.00	1.00

Supplementary Material

Table S3. Summary statistics based on imputation posterior probabilities (2-fields resolution) by African ancestry quartile.

**African Ancestry**	**Q1**	**Q2**	**Q3**	**Q4**
		HLA-A		
Min.	0.15	0.17	0.24	0.13
1st Qu.	0.87	0.86	0.85	0.84
Median	0.94	0.93	0.95	0.95
Mean	0.9	0.89	0.89	0.88
3rd Qu.	0.97	0.98	0.99	0.99
Max.	1.00	1.00	1.00	1.00
		HLA-B		
Min.	0.11	0.06	0.06	0.05
1st Qu.	0.7	0.6	0.55	0.55
Median	0.94	0.89	0.83	0.83
Mean	0.83	0.78	0.74	0.74
3rd Qu.	0.99	0.98	0.97	0.96
Max.	1.00	1.00	1.00	1.00
		HLA-C		
Min.	0.16	0.2	0.12	0.15
1st Qu.	0.97	0.95	0.94	0.92
Median	0.99	0.99	0.99	0.98
Mean	0.96	0.94	0.94	0.92
3rd Qu.	1.00	1.00	1.00	1.00
Max.	1.00	1.00	1.00	1.00
		HLA-DRB1		
Min.	0.29	0.21	0.14	0.2
1st Qu.	0.93	0.91	0.89	0.85
Median	0.98	0.97	0.97	0.95
Mean	0.93	0.92	0.9	0.88
3rd Qu.	0.99	0.99	0.99	0.99
Max.	1.00	1.00	1.00	1.00
HLA-DQB1				
Min.	0.11	0.06	0.08	0.09
1st Qu.	0.56	0.54	0.59	0.57
Median	0.84	0.79	0.85	0.86
Mean	0.75	0.73	0.76	0.76
3rd Qu.	0.98	0.97	0.98	0.98
Max.	1.00	1.00	1.00	1.00

Supplementary Material

Table S4. Number of the HLA alleles in REDOME with low, medium and high resolution in each locus.

**RESOLUTION**	***HLA-A***		***HLA-B***		***HLA-DRB1***		***HLA-C***		***HLA- DQB1***	
	Allele 1	Allele 2	Allele 1	Allele 2	Allele 1	Allele 2	Allele 1	Allele 2	Allele 1	Allele 2
**Low**	1,197,049	1,146,134	1,153,141	1,150,282	1,069,653	1,073,172	3,522	3,350	4,714	4,758
**Medium**	3,462,270	3,476,252	3,265,931	3,261,826	3,223,108	3,283,191	88,011	88,226	145,895	150,374
**High**	64,643	101,576	304,890	311,854	431,201	367,599	30,263	30,220	77,650	73,127
Total (medium-high)			4,723,962 (3,179,618)				121,796		228,259	
A~C~B~DRB1 (medium-high)					121,796 (76,184)					
A~C~B~DRB1 ~DQB1 (medium-high)					106,623 (65,129)					

Supplementary Material

Tables S5. Likelihood of having a low-resolution 6/6, 8/8, 10/10 matched donor by IBGE category

Combination	Group	N	% Match	OR	95% CI	p-value
6/6	Black	1,589	82.8	-	-	-
6/6	Mixed	2,620	84.7	0.849	0.720 - 1.00	0.05
6/6	White	3,544	91.1	0.450	0.379 - 0.532	< 2e-16
8/8	Black	1,589	9.1	-	-	-
8/8	Mixed	2,620	13.6	0.629	0.515 -0.764	3.8e-06
8/8	White	3,544	18.6	0.441	0.366 -0.528	<2e-16
10/10	Black	1,589	7.3	-	-	-
10/10	Mixed	2,620	10.8	0.632	0.507 - 0.782	3.17e-05
10/10	White	3,544	16.9	0.388	0.316 - 0.473	<2e-16

Table S6. Likelihood of having low-resolution 6/6, 8/8, 10/10 matched donor by African genetic ancestry quartile.

Combination	African Quartile	N	% Match	OR	95% CI	p-value
6/6	Q4	2,206	82.00	-	-	-
6/6	Q3	2,205	84.58	1.06	0.85 – 1.31	0.62
6/6	Q2	2,205	90.87	1.95	1.61 – 2.38	1.83e-11
6/6	Q1	2,206	92.10	2.57	2.13 – 3.12	<2e-16
8/8	Q4	2,206	9.6	-	-	-
8/8	Q3	2,205	13.08	1.16	0.99 – 1.34	0.052
8/8	Q2	2,205	17.21	1.66	1.44 – 1.95	5.19e-10
8/8	Q1	2,206	20.36	2.35	1.98 – 2.80	<2e-16
10/10	Q4	2,206	7.3	-	-	-
10/10	Q3	2,205	10.74	1.16	0.99 – 1.35	0.065
10/10	Q2	2,205	15.32	1.87	1.58 – 2.22	8.03e-13
10/10	Q1	2,206	18.52	2.83	2.34 – 3.43	<2e-16

Supplementary Material

Table S7. Number of potential donors between ancestries (Mann-Whitney test with Holm correction for multiple testing).

Ancestry	Loci Set	Group1	Group2	p.value	signif_05
IBGE categories	6/6	Mixed	Black	4.99e-09	TRUE
IBGE categories	6/6	White	Black	1.06e-42	TRUE
IBGE categories	6/6	White	Mixed	2.29e-21	TRUE
IBGE categories	8/8	Mixed	Black	2.80e-01	FALSE
IBGE categories	8/8	White	Black	5.36e-03	TRUE
IBGE categories	8/8	White	Mixed	1.71e-02	TRUE
IBGE categories	10/10	Mixed	Black	4.56e-01	FALSE
IBGE categories	10/10	White	Black	9.02e-02	FALSE
IBGE categories	10/10	White	Mixed	1.10e-01	FALSE
Genetic ancestry	6/6	Q2	Q1	1.57e-05	TRUE
Genetic ancestry	6/6	Q3	Q1	4.68e-20	TRUE
Genetic ancestry	6/6	Q4	Q1	5.26e-56	TRUE
Genetic ancestry	6/6	Q3	Q2	5.73e-07	TRUE
Genetic ancestry	6/6	Q4	Q2	4.61e-32	TRUE
Genetic ancestry	6/6	Q4	Q3	2.81e-11	TRUE
Genetic ancestry	8/8	Q2	Q1	6.10e-01	FALSE
Genetic ancestry	8/8	Q3	Q1	1.83e-01	FALSE
Genetic ancestry	8/8	Q4	Q1	2.12e-04	TRUE

Genetic ancestry	8/8	Q3	Q2	3.88e-01	FALSE
Genetic ancestry	8/8	Q4	Q2	1.90e-03	TRUE
Genetic ancestry	8/8	Q4	Q3	6.29e-02	FALSE
Genetic ancestry	10/10	Q2	Q1	7.78e-01	FALSE
Genetic ancestry	10/10	Q3	Q1	1.82e-01	FALSE
Genetic ancestry	10/10	Q4	Q1	6.89e-03	TRUE
Genetic ancestry	10/10	Q3	Q2	3.00e-01	FALSE
Genetic ancestry	10/10	Q4	Q2	1.79e-02	TRUE
Genetic ancestry	10/10	Q4	Q3	3.00e-01	FALSE
MHC ancestry	6/6	1	0	1.10e-81	TRUE
MHC ancestry	6/6	2	0	1.16e-116	TRUE
MHC ancestry	6/6	2	1	2.80e-15	TRUE
MHC ancestry	8/8	1	0	3.38e-06	TRUE
MHC ancestry	8/8	2	0	2.47e-11	TRUE
MHC ancestry	8/8	2	1	1.34e-04	TRUE
MHC ancestry	10/10	1	0	3.68e-05	TRUE
MHC ancestry	10/10	2	0	3.03e-07	TRUE
MHC ancestry	10/10	2	1	2.45e-03	TRUE

Supplementary Material

Table S8. Proportion of SCD patients eligible for transplantation with at least one match in REDOME, median number of matches and the maximum number of matches at low and medium resolution.

Loci	Low resolution			Medium resolution		
	% at least one match	Median number of matches	Maximum number of matches	% at least one match	Median number of matches	Maximum number of matches
6/6	85.4	11	658	45.8	3	315
8/8	9.6	2	35	3.6	1	14
10/10	8.1	2	18	0.9	1	5

Tables S9. Proportion of individuals with at least one match in REDOME for several dataset combinations.

Dataset	6/6	8/8	10/10
**LOW RESOLUTION**			
REDS-III+EPIGEN	7,024 (87.4%)	1,211 (15.1%)	1,042 (13.0%)
EPIGEN	4,737 (88.8%)	873 (16.4%)	770 (14.4%)
Bambuí+Pelotas	3,934 (89.1%)	712 (16.2%)	622 (14.1%)
REDS-III	2,287 (84.6%)	338 (12.5%)	272 (10.1%)
REDS-Transplant*	356 (85.37%)	40 (9.6%)	34 (8.1%)
**MEDIUM RESOLUTION**			
REDS-III+EPIGEN	4,122 (51.3%)	494 (6.1%)	158 (2.0%)
EPIGEN	2,887 (54.1%)	365 (6.8%)	122 (2.3%)
Bambuí+Pelotas	2,510 (56.8%)	314 (7.1%)	104 (2.3%)
REDS-III	1,235 (45.7%)	129 (4.8%)	36 (1.3%)
REDS-Transplant*	191 (45.8%)	15 (3.6%)	4 (1.0%)

* REDS-SCD patients eligible for transplantation based on the classification by Flor-Park and col. 2019.

Supplementary Material

Table S10. Comparison of the proportion of at least one match between the datasets using the Z- test (p-value)

Dataset I	Dataset II	6/6 z-score (p-value)	8/8 z-score (p-value)	10/10 z-score (p-value)
		LOW RESOLUTION		
REDS transplant*	REDS-III	0.402 (0.689)	-1.696 (0.089)	-1.220 (0.222)
REDS transplant*	EPIGEN	-2.123 (0.034)	-3.646 (<0.0003)	-3.563 (0.0004)
REDS transplant*	Bambuí+Pelotas	-2.295 (0.021)	-3.517 (0.0004)	-3.380 (0.00007)
REDS-III	EPIGEN	-5.357 (<0.0001)	-4.573 (<0.0001)	-4.970 (<0.0001)
REDS-III	Bambuí+Pelotas	-5.519 (<0.0001)	-4.179 (<0.0001)	-5.513 (<0.0001)
		MEDIUM RESOLUTION		
REDS transplant*	REDS-III	0.043 (0.968)	-1.065 (0.289)	-0.629 (0.5287)
REDS transplant*	EPIGEN	-3.281 (0.001)	-2.569 (0.010)	-1.784 (0.075)
REDS transplant*	Bambuí+Pelotas	-4.338 (<0.0001)	-2.723 (0.006)	-1.843 (0.065)
REDS-III	EPIGEN	-7.147 (<0.0001)	-3.651 (0.0002)	-2.915 (0.003)
REDS-III	Bambuí+Pelotas	-9.143 (<0.0001)	-3.963 (0.00008)	-3.017 (0.002)

* REDS-SCD patients eligible for transplantation based on the classification by Flor-Park and col. 2019.

Supplementary Material

Tables S11. Likelihood of having a 6/6, 8/8, 10/10 matched donor by IBGE category for high imputation quality dataset (posterior probability >0.8).

Combination	Resolution	Group	N	% Match	OR	95% CI	p-value
6/6	LOW	Black	374	89.3	-	-	-
6/6	LOW	Mixed	870	90.2	0.904	0.612 - 1.00	0.619
6/6	LOW	White	1,223	95.9	0.356	0.231 - 0.531	2.95e-06
8/8	LOW	Black	374	16.6	-	-	-
8/8	LOW	Mixed	870	18.0	0.902	0.649 -1.00	0.533
8/8	LOW	White	1,223	25.3	0.587	0.431 -0.789	0.0005
10/10	LOW	Black	374	14.2	-	-	-
10/10	LOW	Mixed	870	15.9	0.876	0.617 - 1.00	0.448
10/10	LOW	White	1,223	23.9	0.524	0.377 - 0.715	7.15e-05

Supplementary Material

Table S12. Likelihood of having a 6/6, 8/8, 10/10 matched donor by African genetic ancestry quartile for high imputation quality dataset (posterior probability >0.8).

Combination	Resolution	African Quartile	N	% Match	OR	95% CI	p-value
6/6	LOW	Q4	639	88.9	-	-	-
6/6	LOW	Q3	638	90.1	1.00	0.55 – 1.81	0.99
6/6	LOW	Q2	638	96.4	2.93	1.82 – 4.89	1.72e-05
6/6	LOW	Q1	639	96.4	3.34	2.09 – 5.54	9.86e-07
8/8	LOW	Q4	639	14.7	-	-	-
8/8	LOW	Q3	638	18.0	0.94	0.73 – 1.21	0.643
8/8	LOW	Q2	638	25.5	1.56	1.19 – 2.04	0.001
8/8	LOW	Q1	639	26.6	1.98	1.50 – 2.64	1.88e-06
10/10	LOW	Q4	639	11.6	-	-	-
10/10	LOW	Q3	638	16.6	0.92	0.71 – 1.18	0.507
10/10	LOW	Q2	638	25.7	1.59	1.21 – 2.10	0.001
10/10	LOW	Q1	639	24.1	2.42	1.79 – 3.29	9.71e-09
